# Optimal exercise modality and dose for alleviating depressive symptoms in postmenopausal women: a systematic review and network meta-analysis of randomized controlled trials

**DOI:** 10.3389/fpsyg.2025.1743949

**Published:** 2025-12-11

**Authors:** Peiming Xu, Rui Guo, Lei Yang, Junkai Ding

**Affiliations:** 1 School of Physical Education, Shandong University, Jinan, China; 2 School of Physical Education, Shandong University of Finance and Economics, Jinan, China; 3 School of Physical Education, University of Jinan, Jinan, China

**Keywords:** exercise, depression, postmenopausal women, dose–response, model-based network meta-analysis, RCT

## Abstract

**Objective:**

Although physical exercise is widely recognized as an effective non-pharmacological intervention for depressive symptoms, the relative efficacy of different exercise modalities and the optimal dose for postmenopausal women remain unclear. This study aimed to determine the optimal exercise modality and dose for alleviating depressive symptoms in postmenopausal women through a systematic review and network meta-analysis (NMA), incorporating a model-based network meta-analysis (MBNMA) for dose–response relationships.

**Methods:**

Five major electronic databases (Web of Science, Cochrane Library, PubMed, EBSCO, and Embase) were searched for randomized controlled trials (RCTs) investigating the effects of exercise interventions on depressive symptoms in postmenopausal women. The risk of bias in included studies was assessed using the Cochrane Risk of Bias 2 (RoB 2) tool. The NMA and dose–response MBNMA were conducted using R software.

**Results:**

A total of 33 RCTs involving 2,607 participants were included. The NMA results showed that all four exercise modalities—resistance exercise (RE), mind–body exercise (MBE), aerobic exercise (AE), and combined exercise (CBE)—significantly alleviated depressive symptoms compared to the control group: RE (SMD = −0.90, 95% CI: −1.61 to −0.20), MBE (SMD = −0.75, 95% CI: −1.07 to −0.43), AE (SMD = −0.67, 95% CI: −0.93 to −0.42), and CBE (SMD = −0.63, 95% CI: −1.19 to −0.06). P-score rankings suggested that RE (*p* = 0.774) and MBE (*p* = 0.662) had the greatest therapeutic potential. The dose–response analysis revealed a significant U-shaped relationship, with a minimum effective dose of 183 METs-min/week and an optimal dose around 750 METs-min/week. The therapeutic effect tended to diminish beyond 1,130 METs-min/week. Among the modalities, MBE had the lowest effective dose threshold (164 METs-min/week).

**Conclusion:**

Physical exercise is a highly effective intervention for alleviating depressive symptoms in postmenopausal women, with resistance and mind–body exercises demonstrating the greatest therapeutic potential. The antidepressant effect of exercise follows a U-shaped dose–response relationship, with an optimal dose of approximately 750 METs-min/week. These findings provide robust evidence for clinicians to move beyond generic recommendations and to develop precise, individualized exercise prescriptions tailored to the specific needs and conditions of their patients.

**Systematic review registration:**

PROSPERO, identifier (CRD420251208430).

## 1 Introduction

Menopause represents a significant physiological transition in a woman’s life course (World Health Organization, 2025b), with tens of millions of women entering this stage globally each year. This period is accompanied not only by marked physiological changes but also by a sharp increase in mental health risks. Epidemiological data indicate that postmenopausal women face a significantly elevated risk of developing depression compared to their premenopausal counterparts, with prevalence rates reaching 20 to 40% (Jia et al., 2024; Li et al., 2024). This condition not only severely impairs the quality of life for individual women but also imposes a substantial social and economic burden on global public health systems. Consequently, exploring effective, safe, and easily scalable intervention strategies to address this growing health challenge is of critical practical importance.

Depression is a leading cause of disability worldwide (World Health Organization, 2025a). While traditional treatments such as pharmacotherapy and psychotherapy are effective, their application is often limited by side effects, high costs, or restricted accessibility. Against this backdrop, physical exercise has gained widespread recognition as a non-pharmacological intervention for improving mental health. Numerous systematic reviews and meta-analyses have demonstrated that regular physical exercise can effectively alleviate depressive symptoms in the general adult population (Schuch et al., 2018; Noetel et al., 2024). The underlying biological mechanisms are multifaceted, involving the modulation of monoamine neurotransmitter systems, enhancement of brain-derived neurotrophic factor (BDNF) expression, suppression of hypothalamic–pituitary–adrenal (HPA) axis over-activation, and reduction of systemic inflammation (Craft and Perna, 2004; Basso and Suzuki, 2017).

However, caution is warranted when directly applying these generalized findings to the postmenopausal population. Depressive symptoms in this group are often closely associated with neuroendocrine disturbances stemming from declining estrogen levels and are frequently accompanied by unique physiological degenerative processes such as osteoporosis and sarcopenia (Bondarev et al., 2020; Buckinx and Aubertin-Leheudre, 2022). These factors collectively constitute a complex pathophysiological network, suggesting that interventions targeting this population may require greater specificity. To date, studies have separately investigated the positive effects of different exercise modalities—such as aerobic, resistance, mind–body, and combined exercise—on depressive symptoms in postmenopausal women (Pérez-López et al., 2017; Wang et al., 2025). Nevertheless, this research has often been conducted in isolation, failing to provide a comprehensive comparative framework.

Despite the broad evidence supporting the antidepressant effects of exercise, two critical knowledge gaps hinder its translation into clinical practice and public health guidelines. First, the relative efficacy of different exercise modalities remains unclear. Most existing randomized controlled trials (RCTs) have compared a single exercise modality against a non-exercise control group, while head-to-head studies directly comparing multiple modalities are exceedingly rare. Consequently, clinicians lack high-level evidence to answer the critical question of which type of exercise might be most effective when recommending exercise for postmenopausal women with depression. Traditional pairwise meta-analyses are incapable of synthesizing both direct and indirect evidence to address this question, leading to current guidelines that can only offer generalized exercise recommendations. Second, the dose–response relationship of exercise interventions remains a “black box.” Previous research has largely focused on whether exercise is effective, but evidence is scarce regarding the questions of “How much exercise is needed to be effective?” and “Is there an optimal dose?” Recommendations from organizations like the World Health Organization (WHO), such as 600 METs-min per week, are primarily based on cardiovascular and other physiological health outcomes, and it remains unclear whether they are equally applicable to optimizing mental health in postmenopausal women (World Health Organization, 2020). More importantly, the shape of the dose–response curve for the antidepressant effects of exercise (i.e., whether it is linear, plateaus, or even U-shaped) is poorly understood in the scientific community (Courneya et al., 2017). This uncertainty severely limits the development of precise and individualized “exercise prescriptions.”

Therefore, this study aims to systematically integrate and analyze the existing RCT evidence using advanced statistical methods. This study has two primary objectives. The first is to conduct a Network Meta-Analysis (NMA) that synthesizes both direct and indirect evidence to comprehensively compare the relative efficacy of four major exercise modalities: aerobic exercise (AE), resistance exercise (RE), mind–body exercise (MBE), and combined exercise (CBE), in alleviating depressive symptoms in postmenopausal women. The second objective is to apply a dose–response network meta-analysis (MBNMA) to investigate the relationship between total exercise volume (in METs-min/week) and the improvement of depressive symptoms, aiming to identify the minimum effective dose, the optimal dose, and a potential therapeutic ceiling. We hypothesized that different exercise modalities would exhibit varying levels of efficacy and that the therapeutic effect would not be linear, but rather would follow a relationship with an optimal dosage range.

The remainder of this paper is organized around these objectives. The second section details the methodology for the systematic review and network meta-analysis, including the literature search strategy, inclusion and exclusion criteria, data extraction, and statistical analysis procedures. The third section presents the main findings of the study, including the efficacy rankings from the NMA and the dose–response curve models. The fourth section provides an in-depth discussion of the results, comparing them with existing evidence and elaborating on their clinical implications, as well as the study’s strengths and limitations. Finally, the fifth section concludes the paper with a summary of the findings and provides an outlook on future research directions.

## 2 Methods

### 2.1 Protocol and registration

The protocol for this review was developed in accordance with the principles of the Cochrane Handbook for Systematic Reviews of Interventions and followed the PRISMA Extension Statement for Network Meta-Analyses (PRISMA-NMA) (Higgins and Green, 2008; Hutton et al., 2015). The study was prospectively registered with the International Prospective Register of Systematic Reviews (PROSPERO) under the registration number CRD (CRD420251208430). As this study was based on a re-evaluation of previously published literature, ethical approval and patient informed consent were not required.

### 2.2 Search strategy

To identify the potential effects of different exercise interventions on depressive symptoms in postmenopausal women, a comprehensive literature search was conducted. We systematically searched five key electronic databases: PubMed, Embase, EBSCO, Web of Science, and the Cochrane Library. The search covered the period from the inception of each database to October 2025.

The search strategy combined controlled vocabulary (e.g., Medical Subject Headings, MeSH) with free-text keywords, connected by Boolean operators. Core search terms included “exercise,” “physical activity,” “depression,” “menopause,” and “randomized controlled trial.” The detailed search strategy for each database is provided in Appendix 2. To minimize the risk of missing relevant studies, the reference lists of previously published systematic reviews and meta-analyses were also screened. No language or geographical restrictions were applied to the literature search.

### 2.3 Inclusion and exclusion criteria

The inclusion criteria were defined according to the Population, Intervention, Comparison, Outcome, and Study design (PICOS) framework. Only randomized controlled trials (RCTs) were considered eligible. Studies had to meet all of the following conditions: (1) Population: Participants were women who had entered menopause. Individuals undergoing hormone replacement therapy, diagnosed with any form of cancer, having regular physical exercise habits, or having other comorbidities that could affect cognitive function were excluded. Postmenopausal status was determined based on the inclusion criteria established in each individual randomized controlled trial. While operational definitions varied slightly across studies or were not always explicitly detailed, participants were generally required to have experienced cessation of menstruation for at least 12 months or to present with physiological signs consistent with menopause (e.g., age-appropriate ranges or surgical menopause) (2) Intervention: The exercise intervention had to be one of four predefined types, based on the Physical Activity Guidelines for Americans (Piercy et al., 2018) and previous research (Han et al., 2024): aerobic exercise (AE), combined exercise (CBE), mind–body exercise (MBE), or resistance exercise (RE). The intervention duration was at least four weeks. (3) Comparison: The study had to include a non-exercise control group (e.g., usual care, waitlist, or health education) or, for the purpose of the NMA, compare two different eligible exercise interventions. (4) Outcome: The change in depressive symptoms before and after the intervention had to be measured and reported using a validated scale. (5) Publication Language: The article had to be published in either English or Chinese. Studies were excluded if they met the following criteria: (1) combined exercise with other interventions that could affect depression outcomes, such as psychological or pharmacological therapies; (2) had inaccessible or incomplete data; or (3) were study protocols or conference abstracts.

### 2.4 Study selection

Two reviewers (PM X and R G) independently screened the literature. They first assessed the titles and abstracts of all retrieved records against the predefined inclusion and exclusion criteria and removed duplicate records. Subsequently, the full texts of all potentially eligible articles were subjected to a detailed review to confirm final inclusion. Any disagreements between the two reviewers during the screening process were resolved through discussion or by consulting a third, senior reviewer (JK D).

### 2.5 Data extraction and coding

Data were extracted independently by two reviewers using a standardized data collection form. The following key data points were extracted: demographic characteristics of participants (age), specific details of the intervention (modality, duration, period, and frequency), and information relevant to the risk of bias assessment.

To assess the dose–response characteristics of different exercise modalities, a quantitative measure of weekly exercise volume was calculated. This metric was computed as follows: The Metabolic Equivalent of Task (MET) for a specific activity, multiplied by the duration of a single session, and then multiplied by the weekly frequency. The MET intensity for each exercise modality was determined using the 2024 Compendium of Physical Activities, which provides activity-specific codes for a wide variety of exercises.

To enhance the connectivity of the network and facilitate the dose–response analysis, we estimated the weekly METs-min value for each intervention and categorized them into discrete nodes: 0 (for the non-exercise control group, CON), 300, 600, 900, and 1,200 METs-min/week (Higgins et al., 2012). For studies that did not report MET values, we assigned an average MET value based on the type of intervention described, in accordance with the Compendium of Physical Activities. This process ensured uniformity and comparability in the assessment of exercise intensity across all included trials.

### 2.6 Risk of Bias assessment

Two independent reviewers (PM X and R G) assessed the risk of bias in the included RCTs using the revised Cochrane risk-of-bias tool for randomized trials (RoB 2) (Sterne et al., 2019). This tool uses five domains to reach an overall judgment, classifying studies as having a “low risk of bias,” “some concerns,” or a “high risk of bias.”

### 2.7 Statistical analysis

#### 2.7.1 Network Meta-analysis

To assess the transitivity assumption, we first examined the characteristics of the interventions and the baseline characteristics of participants across trials (Efthimiou et al., 2016). Synthesizing both direct and indirect evidence, an NMA was conducted to improve the precision of comparisons among the different exercise interventions. A network plot was created to visualize the geometry of the included trials. The NMA was performed within a frequentist framework using a random-effects model (Harrer et al., 2021). We ranked the interventions based on their relative effectiveness in alleviating depressive symptoms using P-scores (Rücker and Schwarzer, 2015). A P-score ranges from 0 to 1, with a higher value indicating a greater probability of being the best intervention. Overall network heterogeneity was quantified using τ^2^ and the I^2^ statistic, and prediction intervals were included in the forest plots to visualize the extent of heterogeneity. The node-splitting method was used to detect local inconsistency in the network, while a design-by-treatment interaction model was used to assess global inconsistency (Dias et al., 2010; Higgins et al., 2012). The NMA was performed using the ‘netmeta’ package in R.”Multi-arm trials (studies with more than two relevant intervention groups) were handled using the standard adjustment method within the “netmeta” package, which automatically accounts for the correlation of effect sizes between comparisons within the same study.”

#### 2.7.2 Dose–response network Meta-analysis

A model-based network meta-analysis (MBNMA) within a random-effects Bayesian framework was employed to investigate the dose–response relationship between different exercise modalities and depressive symptoms in postmenopausal women (Mawdsley et al., 2016). We first verified the assumptions of transitivity (Higgins et al., 2012), consistency (Wheeler et al., 2010), and network connectivity (Ter Veer et al., 2019) (see Appendix 9). Due to the variety of depression scales used, the standardized mean difference (SMD) with 95% credible intervals (CrI) was used as the measure of effect size. Among the alternative dose–response models considered (e.g., Emax, restricted cubic splines, and non-parametric models), a second-order (quadratic) polynomial model demonstrated the best fit based on the Deviance Information Criterion (DIC), between-study variance, parameter estimates, and residual error (Evans, 2019) (Appendix 10). This quadratic random-effects framework is particularly adept at modeling non-linear relationships, especially in contexts involving duration and intensity dependencies. By extracting the beta coefficients from this model, we identified the exercise dose threshold required to induce a significant change in depression scores and generated dose–response plots. Subsequently, interventions were ranked based on the sensitivity of symptom improvement to their modality and dose (Mawdsley et al., 2016). The MBNMA and dose–response analyses were conducted using the ‘MBNMAdose’ package (version 4.3.1) in R, and the dose–response plots were generated and optimized using the ‘ggplot2’ package.

#### 2.7.3 Sensitivity analysis and network Meta-regression

To assess the robustness of the findings and explore potential effect modifiers, we conducted additional sensitivity and meta-regression analyses. First, to address measurement uncertainty in unsupervised settings, we performed a sensitivity analysis on the dose–response relationship by restricting the dataset to studies with full or hybrid supervision. Second, network meta-regression was utilized to evaluate the influence of supervision status and mean participant age on the intervention efficacy.

## 3 Results

### 3.1 Study selection and characteristics

**Figure 1:**
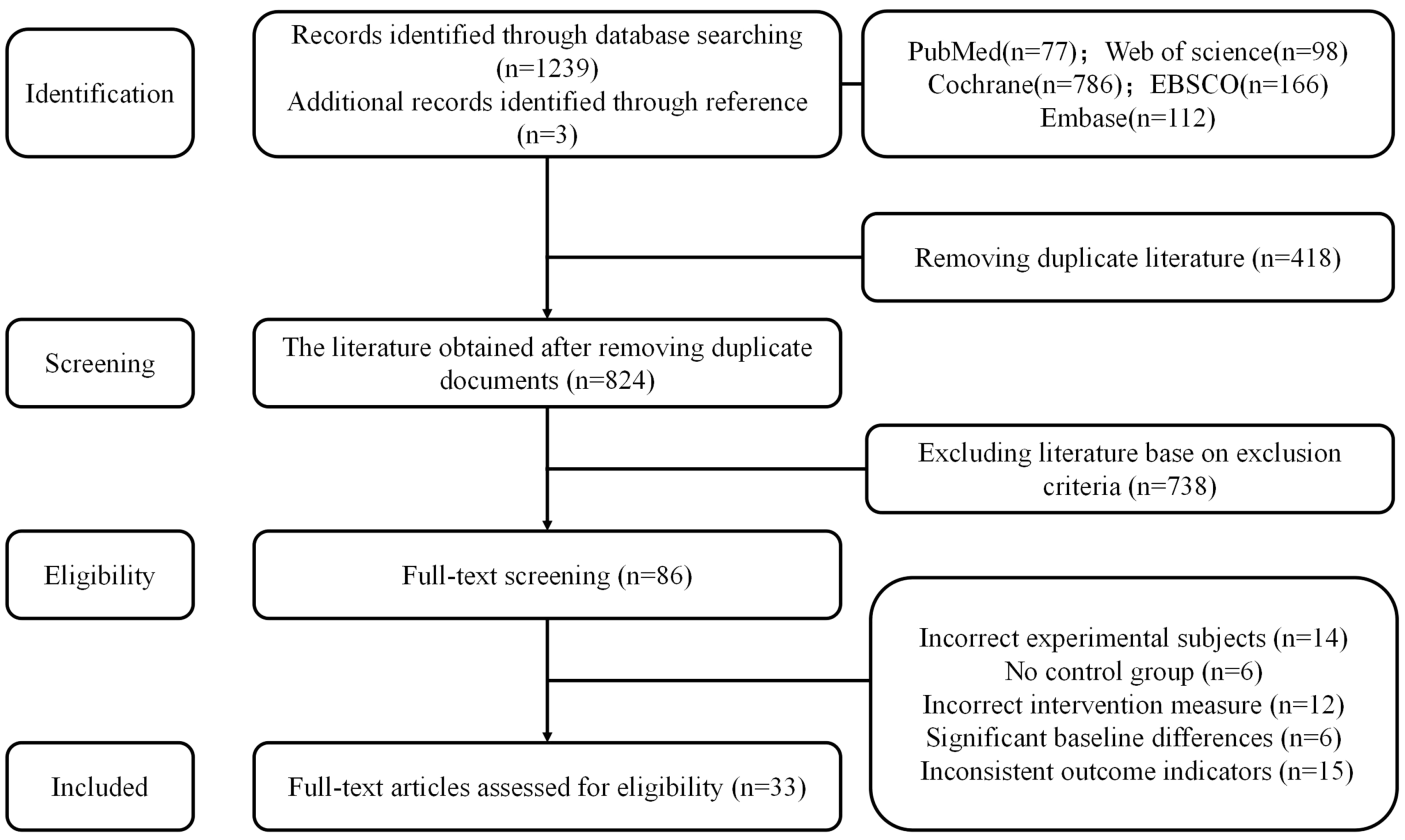
PRISMA flowchart of included studies.

**Figure 2:**
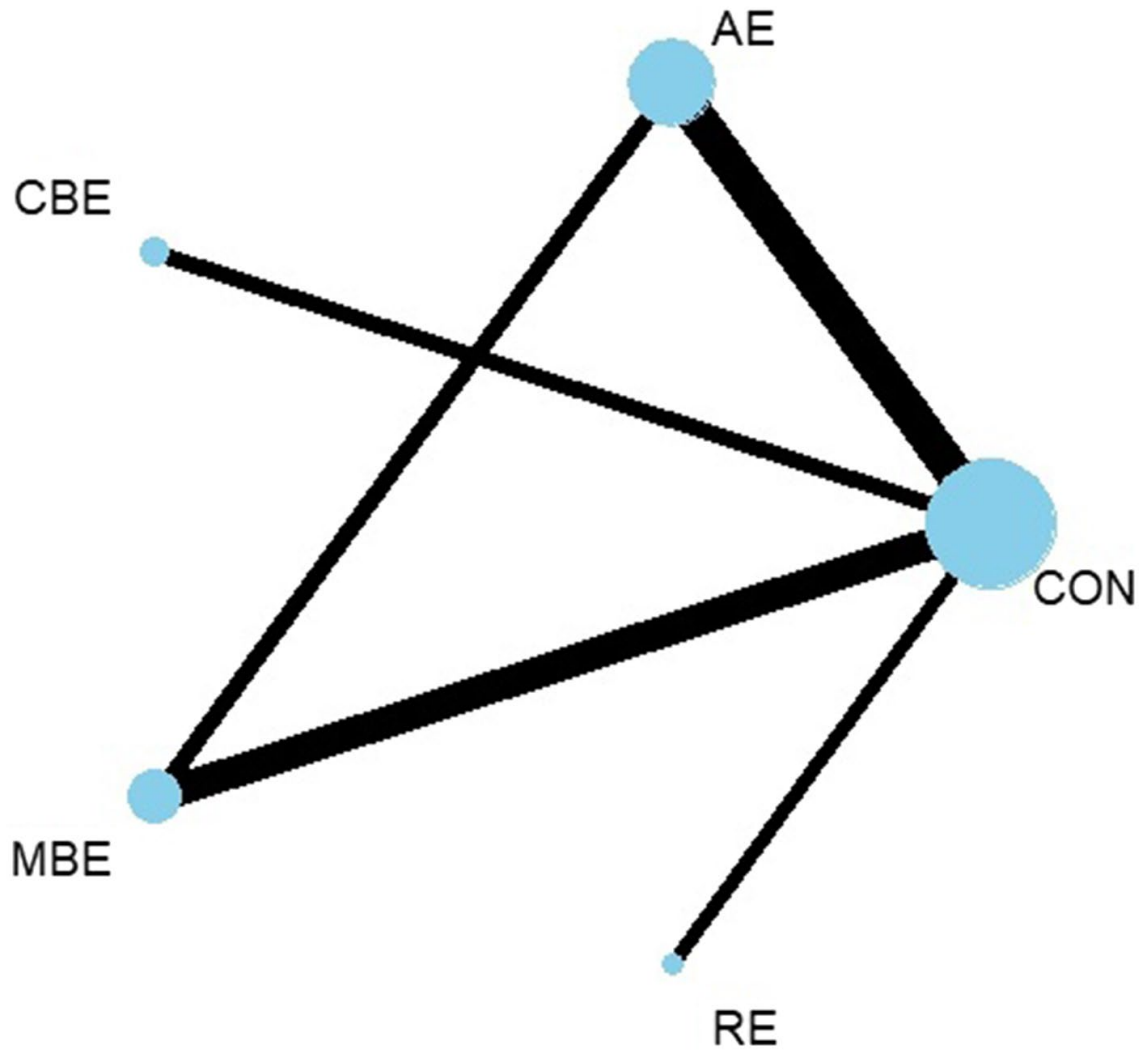
Network plot of comparisons for all studies included in the network meta-analysis.

The initial electronic search yielded 1,239 studies. After removing 418 duplicate entries, we screened the titles and abstracts of 824 articles. From this screening, the full texts of 86 studies were assessed for eligibility. Following a detailed review, 53 trials were excluded for various reasons. Ultimately, 33 trials were included in our meta-analysis (Figure 1). The network diagram of all paired comparisons is shown in Figure 2. Across the 33 included studies, a total of 2,607 participants were evaluated. The intervention durations ranged from 3 to 48 weeks, with frequencies varying from 3 to 5 sessions per week. The included studies examined the effects of different exercise interventions: 18 studies investigated AE, 4 investigated CBE, 12 investigated MBE, and 3 investigated RE. Detailed characteristics of all included studies are available in Appendix 1. Depressive symptoms were assessed using validated scales, with the most frequently utilized instruments being the Beck Depression Inventory (BDI), the Hamilton Depression Rating Scale (HAMD), and the Geriatric Depression Scale (GDS). Based on the baseline scores reported in the included studies, participants generally presented with mild-to-moderate depressive symptoms or were identified as being at risk for depression.

### 3.2 Risk of Bias and certainty of evidence

Among the 33 studies included in this meta-analysis, three were assessed as having a high risk of bias, 24 had some concerns, and six had a low risk of bias. Detailed information on the risk of bias assessment is presented in Appendix 3. Due to issues of uncertainty and potential bias, the overall quality of evidence for this review was rated as very low to moderate. A summary of the findings is provided in Appendix 7.

### 3.3 Network Meta-analysis

**Figure 3:**
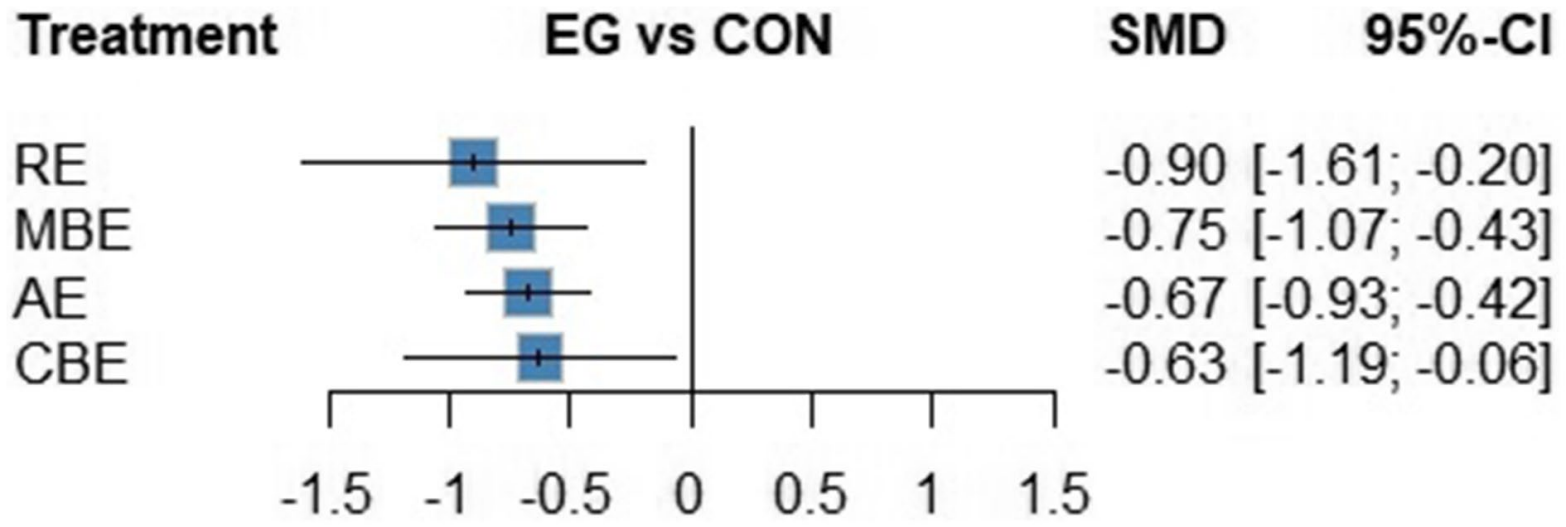
Random-effects meta-analysis for exercise versus control on the depression network.

A total of 33 studies involving 2,607 participants were included in the NMA comparing the efficacy of AE, CBE, MBE, and RE for improving depressive symptoms. Compared with the control group, all four exercise modalities significantly reduced depression scores: AE (SMD = −0.67, 95% CI: −0.93 to −0.42), CBE (SMD = −0.63, 95% CI: −1.19 to −0.06), MBE (SMD = −0.75, 95% CI: −1.07 to −0.43), and RE (SMD = −0.90, 95% CI: −1.61 to −0.20) (Figure 3).

**Figure 4:**
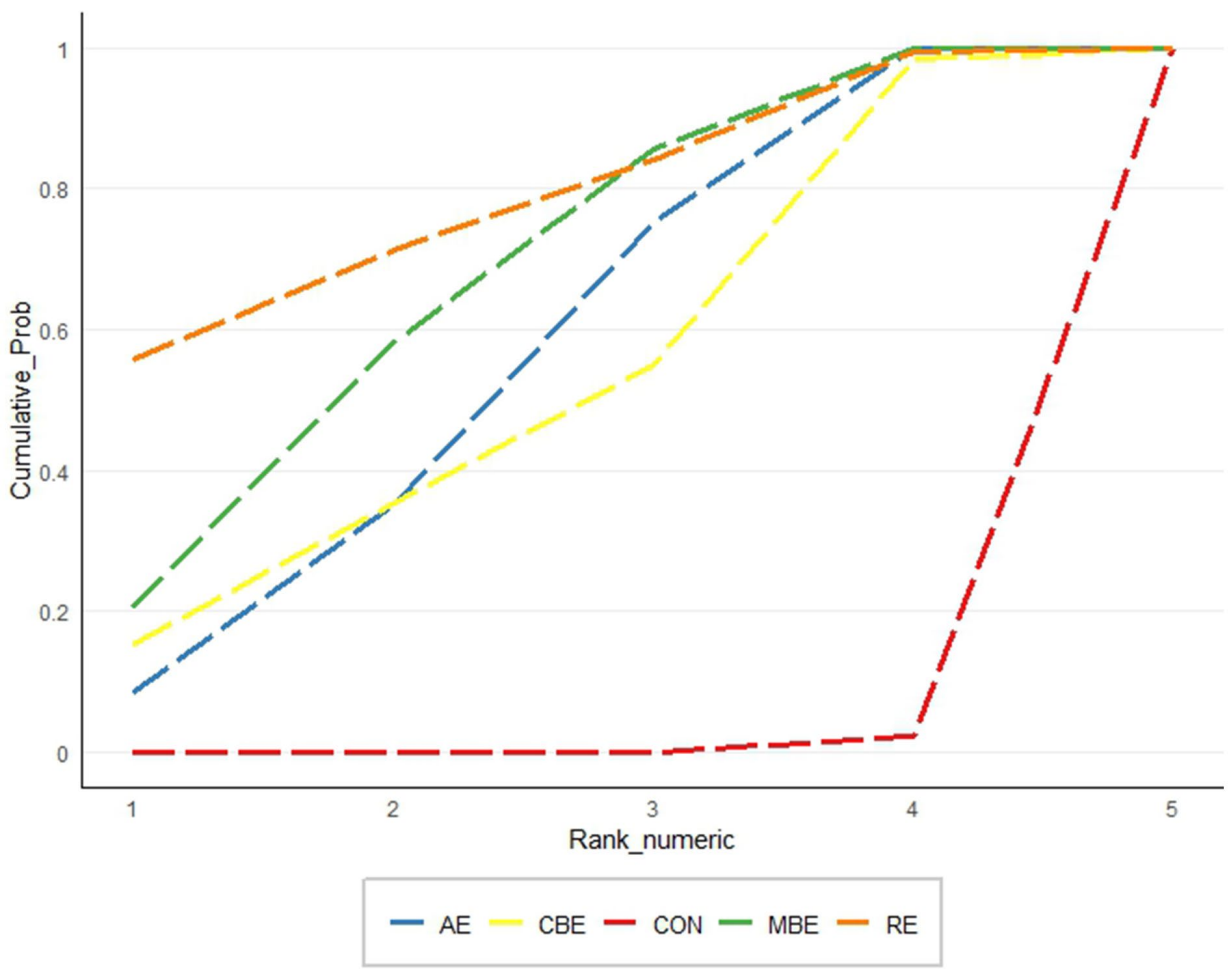
Cumulative ranking probability plots for depression.

Based on P-score rankings, RE was ranked first (*p* = 0.774), followed by MBE (*p* = 0.662), AE (*p* = 0.543), and CBE (*p* = 0.516). No significant differences were found in the direct comparisons of efficacy between the four exercise modalities (Figure 4).

The network heterogeneity was low (τ^2^ = 0.2446, *I*^2^ = 0.8%). The global Q-statistic for inconsistency was 165.37, and no significant local inconsistency was detected across the five treatment comparisons, indicating high consistency between direct and indirect evidence. A sensitivity analysis, which involved removing the three studies with the highest risk of bias, confirmed that the intervention rankings remained unchanged, demonstrating the robustness of the results (Table 1).

**Table 1:** Comparative effectiveness results of interventions in network meta-analysis and sensitivity analysis.

AE	0.23	−0.04	0.11	**−0.67**
P-score: 0.543	(−0.54, 0.99)	(−0.68, 0.59)	(−0.28, 0.50)	**(−0.94, −0.40)**
0.23 (−0.52, 0.98)	RE P-score: 0.774	−0.27 (−1.19, 0.65)	−0.11 (−0.90, 0.68)	**−0.90 (−1.61, −0.19)**
−0.04 (−0.67, 0.58)	−0.27 (−1.18, 0.63)	CBE P-score: 0.516	0.16 (−0.51, 0.82)	**0.63 (−1.20, −0.05)**
0.08 (−0.30, 0.45)	−0.15 (−0.93,0.62)	0.12 (−0.53,0.77)	MBE P-score: 0.662	**−0.79 (−1.12, −0.45)**
**−0.67 (−0.93, −0.42)**	**−0.90 (−1.61, −0.20)**	**−0.63 (−1.19, −0.06)**	**−0.75 (−1.07, −0.43)**	CON P-score:0.005

The lower left corner shows the mesh analysis results, and the upper right corner shows the sensitivity analysis results.

### 3.4 Dose–response relationship

**Figure 5:**
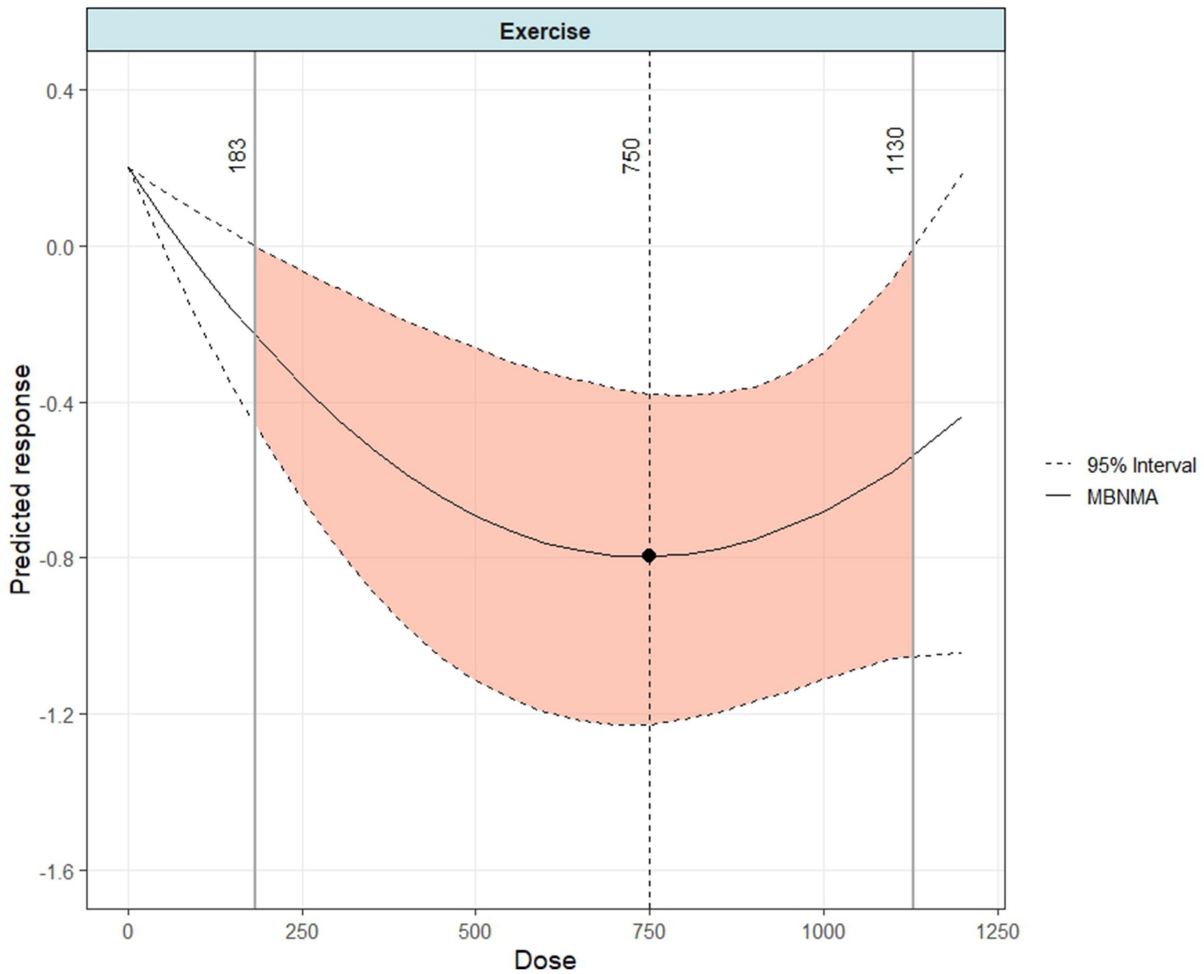
Dose–response relationship between overall physical activity dose and changes in depressive symptoms in postmenopausal women.

A distinct U-shaped relationship was observed between the total exercise dose and depression scores (Figure 5). A statistically significant dose–response effect began at 183 METs-min/week (SMD = −0.23; 95% CrI: −0.45 to −0.01). The model predicted a peak effect at approximately 750 METs-min/week (SMD = −0.80; 95% CrI: −1.21 to −0.39), with the therapeutic benefit appearing to diminish beyond a dose of approximately 1,130 METs-min/week (SMD = −0.53; 95% CrI: −1.03 to −0.03). At the WHO-recommended level of 600 METs-min/week (World Health Organization, 2020), the predicted effect was an SMD of −0.75 (95% CrI: −1.18 to −0.33). These values provide a reference framework for comparing the efficacy of different exercise doses.

**Figure 6:**
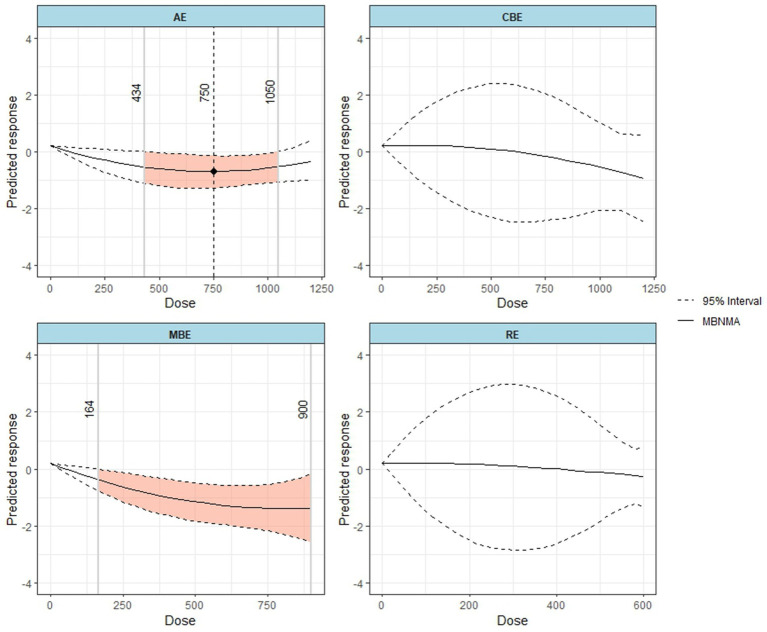
Dose–response relationships between different types of physical activity and exercise doses and changes in depressive symptoms.

As shown in Figure 6, the effects of different exercise modalities on alleviating depressive symptoms varied considerably. Only aerobic exercise (AE) and mind–body exercise (MBE) demonstrated clear U-shaped dose–response curves. The model estimated that the minimum effective dose for AE was 434 METs-min/week (SMD = −0.54; 95% CrI: −1.10 to −0.01). Dividing this value by an average MET value of 6 for aerobic activities suggests that a minimum of approximately 70 min of AE per week is required to produce a significant effect. In contrast, mind–body exercise (MBE) showed a lower effective dose threshold, with a significant effect beginning at 164 METs-min/week (SMD = −0.37; 95% CrI: −0.74 to −0.02). Dividing this value by an average MET value of 3.5 for mind–body activities suggests that a minimum of approximately 45 min of MBE per week is needed for a significant effect.

Further analysis indicated that combined exercise (CBE) and resistance exercise (RE) showed a negative non-linear association with depression severity. However, it is important to acknowledge that these modality-specific curves, particularly for RE, were derived from a limited number of studies (*n* = 3), resulting in wider credible intervals and reduced precision of the estimates. At the WHO-recommended minimum activity level (600 METs-min/week), MBE had the best-predicted efficacy (SMD = −1.28; 95% CrI: −1.97 to −0.58), whereas CBE was the least effective (SMD = 0.03; 95% CrI: −2.27 to 2.37). In terms of dose-ranking, MBE achieved its optimal effect at a dose of 900 METs-min/week. The rankings of all interventions for improving depressive symptoms are presented in Table 2.

**Table 2:** Predictions ranking (from best to worst).

Rank	Intervention-dose	Mean	95%CI
1	MBE_900	−1.36	(−2.52, −0.15)
2	MBE_680	−1.35	(−2.06, −0.57)
3	MBE_450	−1.08	(−1.74, −0.42)
4	CBE_1,200	−0.91	(−2.42, 0.64)
5	AE_600	−0.66	(−1.26, −0.10)
6	AE_900	−0.65	(−1.14, −0.14)
7	MBE_220	−0.55	(−1.03, −0.08)
8	AE_300	−0.38	(−0.84, 0.05)
9	AE_1,200	−0.34	(−0.98, 0.38)
10	CBE_900	−0.31	(−2.20, 1.43)
11	RE_600	−0.27	(−1.26, 0.72)
12	RE_450	−0.10	(−2.24, 2.05)
13	RE_300	0.03	(−2.79, 2.80)
14	CBE_600	0.07	(−2.30, 2.35)
15	RE_150	0.13	(−1.97, 2.20)
16	CBE_300	0.24	(−1.56, 1.98)

### 3.5 Sensitivity analysis and network Meta-regression

To verify the stability of the U-shaped dose–response relationship, we analyzed the subset of studies involving strictly supervised interventions. The sensitivity analysis confirmed that the U-shaped trajectory remained robust even when restricted to supervised trials, with the optimal dose range consistent with the primary analysis (Appendix 13).

Furthermore, network meta-regression was conducted to examine potential moderators. The results indicated that supervision status did not significantly moderate the antidepressant effects (Coefficient = 0.264, *p* = 0.449), suggesting that the inclusion of studies with estimated METs did not introduce significant bias. Similarly, mean participant age showed no significant association with treatment efficacy (Coefficient = 0.0124, *p* = 0.424) (Appendix 13), indicating that exercise interventions retain their efficacy across different stages of the postmenopausal period.

## 4 Discussion

### 4.1 Main findings

This study provides several key findings with critical clinical implications for exercise intervention strategies aimed at alleviating depressive symptoms in postmenopausal women. First, our network meta-analysis confirms that all major exercise modalities, including aerobic (AE), resistance (RE), mind–body (MBE), and combined (CBE) exercise, significantly reduce depressive symptoms after a minimum duration of four weeks. In terms of efficacy ranking, resistance exercise (*p* = 0.774) and mind–body exercise (*p* = 0.662) demonstrated the greatest therapeutic potential. Second, this study is the first to reveal, through a dose–response network meta-analysis, a distinct U-shaped relationship between the overall exercise volume and its therapeutic effect. The minimum effective dose was identified as 183 METs-min/week, with the optimal dose occurring at approximately 750 METs-min/week, beyond which the effect tended to diminish (above 1,130 METs-min/week). Third, we further identified that the dose–response curves differ among modalities: AE and MBE exhibited significant U-shaped curves, whereas RE and CBE showed a negative non-linear association. Given the sparse data available for these modalities at certain dose ranges, these specific patterns should be interpreted with caution. Finally, MBE had the lowest effective dose threshold (164 METs-min/week), offering a viable therapeutic option for individuals with limited physical capacity. In summary, these findings offer a new perspective for developing future exercise guidelines aimed at improving depressive symptoms and alleviating the associated burden in postmenopausal women.

### 4.2 Comparison with existing evidence

The findings of this study both corroborate and extend the existing scientific literature, particularly in the refined analysis of specific populations, exercise modalities, and dosages (Schuch and Stubbs, 2019).

Our research reaffirms the efficacy of exercise for depressive symptoms, a conclusion consistent with numerous large-scale meta-analyses in the general adult population (Pearce et al., 2022; Noetel et al., 2024). The unique contribution of our study lies in its specific focus on postmenopausal women. This is a distinct population experiencing significant endocrine changes, often accompanied by musculoskeletal degeneration, which may contribute to a more complex pathophysiological mechanism for depression (Dunn et al., 2005; Clayton and Ninan, 2010). Therefore, our study provides more precise evidence supporting the application of exercise interventions in this specific demographic.

A noteworthy finding is the superior performance of resistance exercise (RE) in our efficacy rankings (Gordon et al., 2018). This result suggests that exercise recommendations should not be limited to the traditionally emphasized aerobic activities (Singh et al., 2023). Although previous research has confirmed the positive impact of resistance training on depressive symptoms in older adults (Gordon et al., 2018; Cunha et al., 2024), it has generally failed to clarify its relative efficacy compared to other modalities within a comprehensive network meta-analysis. By integrating a broader evidence network, our NMA provides a quantitative basis for the potential of RE in alleviating depressive symptoms in postmenopausal women. This efficacy may stem from its unique physiological and psychological mechanisms. Physiologically, resistance training effectively improves insulin resistance, reduces systemic low-grade inflammation associated with depression (Kraemer et al., 2002), and may more effectively stimulate the synthesis of brain-derived neurotrophic factor (BDNF) and insulin-like growth factor-1 (IGF-1), which are crucial for maintaining neuroplasticity and hippocampal function. Psychologically, resistance training offers concrete and quantifiable feedback on progress (e.g., increased load), and this objective confirmation of one’s capabilities can effectively enhance self-efficacy, directly counteracting core depressive symptoms such as helplessness and low self-esteem (Gordon et al., 2018).

The U-shaped dose–response relationship identified in our study refines the conventional linear model of the antidepressant effects of exercise. Previous research has largely focused on establishing a “minimum effective dose” and has tended to assume a “more is better” hypothesis, with insufficient exploration of an “optimal dose” or potential “plateau or attenuation phase” (Pearce et al., 2022). Our analysis clearly delineates that the antidepressant effect peaks at approximately 750 METs-min/week, with no additional benefits observed beyond 1,130 METs-min/week (Tian et al., 2024). The plateauing and subsequent decline of the curve at higher doses provide dose-dependent evidence for the negative psychological effects of “overtraining” (Kreher and Schwartz, 2012). The underlying mechanism may relate to chronic physiological stress; for example, sustained high-intensity training can lead to persistent activation of the hypothalamic–pituitary–adrenal (HPA) axis, dysregulated cortisol levels, and excessive production of inflammatory cytokines, all of which are established biological underpinnings of depression (Heijnen et al., 2016). Our findings suggest that clinical practice should shift from merely encouraging increased exercise volume to recommending an optimal dose to maximize benefits and mitigate potential risks. Furthermore, regarding the composition of the exercise dose, it is important to note that our MBNMA model utilizing METs-min/week inherently integrates exercise intensity (calculated as Intensity [MET value] × Duration × Frequency). Although a categorical subgroup analysis (low vs. moderate vs. high) was not feasible due to the predominance of moderate-intensity protocols (~85%) in the included literature, the observed U-shaped relationship suggests that total physiological load is the determining factor (Kandola et al., 2019; Saeed et al., 2019; Zou et al., 2018). Excessive load, whether driven by high intensity or prolonged duration, may trigger stress responses that counteract antidepressant benefits. Additionally, a critical distinction must be made between the acute therapeutic phase and long-term maintenance. Among the included trials, only a small fraction reported follow-up data beyond the intervention period. A qualitative synthesis of these limited data yields mixed results: while some aerobic interventions demonstrated sustained benefits up to 12 months (Blumenthal et al., 1991), others engaging in high-impact exercise observed a regression of benefits shortly after cessation (Sen et al., 2020). This indicates that the “optimal dose” of 750 METs-min/week identified in this study primarily reflects the requirement for active symptom alleviation. The “maintenance dose” required to prevent relapse remains an unaddressed gap in the current literature.

Our analysis of mind–body exercise (MBE) also offers a new perspective. MBE required the lowest dose (164 METs-min/week) to achieve a statistically significant effect. This indicates that its therapeutic mechanism may not be limited to physical activity alone (Xu et al., 2024). Practices like yoga and Tai Chi integrate breath regulation, attentional focus, and interoceptive awareness, elements that are core techniques in established psychological interventions such as mindfulness-based cognitive therapy (MacKenzie and Kocovski, 2016). Through these practices, individuals learn to observe their emotions and bodily sensations non-judgmentally, which can improve autonomic nervous system function and enhance emotional regulation (Pascoe et al., 2017). Thus, the antidepressant effect of MBE is likely a composite of physiological activity and cognitive-behavioral training. This also explains why MBE can produce significant therapeutic effects at a relatively low physical load, making it an ideal option for postmenopausal women with physical limitations or low initial adherence to exercise.

### 4.3 Clinical implications

The findings from this dose–response analysis hold significant clinical implications. First, the results underscore the variations in efficacy across different exercise modalities for alleviating depressive symptoms in postmenopausal women. Specifically, resistance exercise (RE) and mind–body exercise (MBE) demonstrated substantial therapeutic potential. These findings provide a foundation for clinicians to develop personalized treatment strategies tailored to individual patient profiles, thereby accommodating their unique physical conditions, comorbidities, and personal preferences.

Second, the optimal exercise volume associated with antidepressant efficacy identified in this study (approximately 750 METs-min/week) exceeds the minimum physical activity threshold recommended by the World Health Organization (WHO) for the general adult population (600 METs-min/week). This estimated optimal dose can be translated into specific, actionable exercise prescriptions. For instance, this equates to approximately 188 min of moderate-intensity resistance training (4.0 METs), 125 min of moderate-intensity aerobic exercise (6.0 METs), or 250 min of yoga practice (3.0 METs) per week. This practical insight is highly significant, as it provides both patients and clinicians with a clear target dosage aimed at maximizing therapeutic outcomes.

Furthermore, the study revealed that a relatively low dose of mind–body exercise (164 METs-min/week) is sufficient to significantly improve depressive symptoms, corresponding to only about 55 min of yoga or Tai Chi practice per week. This finding suggests that mind–body exercises may exert their antidepressant effects through distinct mechanisms and pathways. This is particularly relevant for postmenopausal women who struggle with high-intensity exercise due to physical pain, severe fatigue, or lack of motivation, as they can achieve antidepressant benefits through this highly accessible exercise volume. Finally, the results confirm the positive effects of combined exercise interventions on alleviating depressive symptoms. This provides a scientific basis for developing holistic exercise regimens that integrate aerobic activity with muscle-strengthening training, thereby offering a multifaceted approach to improving the physical and mental well-being of postmenopausal women.

### 4.4 Clinical heterogeneity and comorbidities

Our meta-regression demonstrated that age does not limit the efficacy of exercise (*p* = 0.424), suggesting benefits for both early and late postmenopausal women. Regarding comorbidities, although quantitative analysis was limited, a qualitative review highlights the need for personalization. For instance, Afonso et al. (2012) showed that yoga benefits women with insomnia, while Sen et al. (2020) found high-impact exercise effective for those with osteopenia. Future prescriptions should tailor the exercise modality to the patient’s specific comorbid profile.

### 4.5 Strengths and limitations

The primary strength of this study lies in its advanced and comprehensive methodology. We are the first to apply a Bayesian dose–response network meta-analysis (MBNMA) to integrate both direct and indirect evidence on four major exercise modalities, allowing us not only to compare their relative efficacy but also to precisely map their dose–response curves (Gallardo-Gómez et al., 2023). This combined approach enabled a detailed examination of the dose–response relationship between different exercise types and depression in postmenopausal women, thereby providing stronger clinical evidence for our findings. Second, we systematically compared the relative efficacy of various active interventions using direct, indirect, and network estimates to identify the minimum dose of MBE needed to enhance treatment outcomes for depression.

However, this study also has some limitations. First, there was heterogeneity among the included studies regarding intervention details, control group designs, and depression assessment tools. Although a random-effects model was used to account for some of this variation, its influence cannot be entirely dismissed. Second, the MET values for exercise volume were estimated based on standard compendiums in some studies, which may not perfectly reflect actual energy expenditure; therefore, these findings should be interpreted with caution. Third, while we assessed and adjusted for publication bias, the possibility of selective reporting and the exclusion of unpublished negative-result studies cannot be completely ruled out. Fourth, the high homogeneity of exercise intensity across the included studies (predominantly moderate intensity) prevented a granular quantitative analysis of low- versus high-intensity interventions. While our MET-based model accounts for intensity mathematically, specific recommendations for high-intensity interval training (HIIT) or low-intensity restorative practices require further investigation. The scarcity of long-term follow-up data in the existing RCTs limits our ability to assess the durability of the antidepressant effects or to determine an optimal ‘maintenance dose’ for postmenopausal women. Finally, given that the overall certainty of evidence ranged from very low to moderate and several included trials raised some concerns regarding risk of bias, these findings should be applied with caution in clinical practice and guideline development.

## 5 Conclusion

This comprehensive review of existing evidence demonstrates that physical exercise is a highly effective non-pharmacological intervention for alleviating depressive symptoms in postmenopausal women. Among the various exercise modalities, resistance and mind–body exercises showed the greatest therapeutic potential. Furthermore, this study revealed a U-shaped dose–response relationship for exercise interventions, identified an optimal dose of approximately 750 METs-min/week, and found that mind–body exercise has the lowest effective dose threshold. These findings provide a robust evidence base for clinicians to move beyond generic recommendations and to develop precise, individualized exercise prescriptions tailored to the specific needs and conditions of their patients, thereby maximizing therapeutic outcomes and reducing the burden of depression in the postmenopausal population.

## Data Availability

The original contributions presented in the study are included in the article/Supplementary material, further inquiries can be directed to the corresponding author.

## References

[ref9001] Afonso R. F. Hachul H. Kozasa E. H. Oliveira D. de S. Goto V. . (2012). Yoga decreases insomnia in postmenopausal women: a randomized clinical trial. Menopause 19, 186–193. doi: 10.1097/gme.0b013e318228225f 22048261

[ref1] Basso J. C. Suzuki W. A. (2017). The effects of acute exercise on mood, cognition, neurophysiology, and neurochemical pathways: a review. Brain Plast 2, 127–152. doi: 10.3233/BPL-160040, 29765853 PMC5928534

[ref9002] Blumenthal J. A. Emery C. F. Madden D. J. Schniebolk S. Walsh-Riddle M. George L. K. . (1991). Long-term effects of exercise on psychological functioning in older men and women. J Gerontol 46, P352–361. doi: 10.1093/geronj/46.6.p352 1940092

[ref2] Bondarev D. Sipilä S. Finni T. Kujala U. M. Aukee P. Laakkonen E. K. . (2020). The role of physical activity in the link between menopausal status and mental well-being. Menopause 27, 398–409. doi: 10.1097/GME.0000000000001490, 32049927 PMC7147406

[ref3] Buckinx F. Aubertin-Leheudre M. (2022). Sarcopenia in menopausal women: current perspectives. Int. J. Women's Health 14, 805–819. doi: 10.2147/IJWH.S340537, 35769543 PMC9235827

[ref4] Clayton A. H. Ninan P. T. (2010). Depression or menopause? Presentation and management of major depressive disorder in perimenopausal and postmenopausal women. Prim. Care Comp. J. Clin. Psychiatry 12:PCC.08r00747. doi: 10.4088/PCC.08r00747blu, 20582297 PMC2882813

[ref5] Courneya K. S. McNeil J. O’Reilly R. Morielli A. R. Friedenreich C. M. (2017). Dose-response effects of aerobic exercise on quality of life in postmenopausal women: results from the breast Cancer and exercise trial in Alberta (BETA). Ann. Behav. Med. 51, 356–364. doi: 10.1007/s12160-016-9859-8, 27837524

[ref6] Craft L. L. Perna F. M. (2004). The benefits of exercise for the clinically depressed. Prim. Care Comp. J. Clin. Psychiatry 6, 104–111. doi: 10.4088/pcc.v06n0301, 15361924 PMC474733

[ref7] Cunha P. M. Werneck A. O. Santos L. D. Oliveira M. D. Zou L. Schuch F. B. . (2024). Can resistance training improve mental health outcomes in older adults? A systematic review and meta-analysis of randomized controlled trials. Psychiatry Res. 333:115746. doi: 10.1016/j.psychres.2024.115746, 38281452

[ref8] Dias S. Welton N. J. Caldwell D. M. Ades A. E. (2010). Checking consistency in mixed treatment comparison meta-analysis. Stat. Med. 29, 932–944. doi: 10.1002/sim.3767, 20213715

[ref9] Dunn A. L. Trivedi M. H. Kampert J. B. Clark C. G. Chambliss H. O. (2005). Exercise treatment for depression: efficacy and dose response. Am. J. Prev. Med. 28, 1–8. doi: 10.1016/j.amepre.2004.09.003, 15626549

[ref10] Efthimiou O. Debray T. P. A. van Valkenhoef G. Trelle S. Panayidou K. Moons K. G. M. . (2016). GetReal in network meta-analysis: a review of the methodology. Res. Synth. Methods 7, 236–263. doi: 10.1002/jrsm.1195, 26754852

[ref11] Evans N. J. (2019). Assessing the practical differences between model selection methods in inferences about choice response time tasks. Psychon. Bull. Rev. 26, 1070–1098. doi: 10.3758/s13423-018-01563-9, 30783896 PMC6710222

[ref12] Gallardo-Gómez D. Del Pozo-Cruz J. Pedder H. Alfonso-Rosa R. M. Álvarez-Barbosa F. Noetel M. . (2023). Optimal dose and type of physical activity to improve functional capacity and minimise adverse events in acutely hospitalised older adults: a systematic review with dose-response network meta-analysis of randomised controlled trials. Br. J. Sports Med. 57, 1272–1278. doi: 10.1136/bjsports-2022-106409, 37536984

[ref13] Gordon B. R. McDowell C. P. Hallgren M. Meyer J. D. Lyons M. Herring M. P. (2018). Association of Efficacy of resistance exercise training with depressive symptoms: Meta-analysis and Meta-regression analysis of randomized clinical trials. JAMA Psychiatry 75, 566–576. doi: 10.1001/jamapsychiatry.2018.0572, 29800984 PMC6137526

[ref14] Han B. Duan Y. Zhang P. Zeng L. Pi P. Chen J. . (2024). Effects of exercise on depression and anxiety in postmenopausal women: a pairwise and network meta-analysis of randomized controlled trials. BMC Public Health 24:1816. doi: 10.1186/s12889-024-19348-2, 38977980 PMC11229230

[ref15] Harrer M. Cuijpers P. Furukawa T. Ebert D. (2021). Doing Meta-analysis with R: A hands-on guide. New York: Chapman and Hall/CRC.

[ref16] Heijnen S. Hommel B. Kibele A. Colzato L. S. (2016). Neuromodulation of aerobic exercise—a review. Front. Psychol. 6:1890. doi: 10.3389/fpsyg.2015.01890, 26779053 PMC4703784

[ref17] Higgins J. P. Green S. (2008). Cochrane handbook for systematic reviews of interventions. Hoboken: John Wiley & Sons.

[ref18] Higgins J. P. T. Jackson D. Barrett J. K. Lu G. Ades A. E. White I. R. (2012). Consistency and inconsistency in network meta-analysis: concepts and models for multi-arm studies. Res. Synth. Methods 3, 98–110. doi: 10.1002/jrsm.1044, 26062084 PMC4433772

[ref19] Hutton B. Salanti G. Caldwell D. M. Chaimani A. Schmid C. H. Cameron C. . (2015). The PRISMA extension statement for reporting of systematic reviews incorporating network meta-analyses of health care interventions: checklist and explanations. Ann. Intern. Med. 162, 777–784. doi: 10.7326/M14-2385, 26030634

[ref20] Jia Y. Zhou Z. Xiang F. Hu W. Cao X. (2024). Global prevalence of depression in menopausal women: a systematic review and meta-analysis. J. Affect. Disord. 358, 474–482. doi: 10.1016/j.jad.2024.05.051, 38735578

[ref21] Kandola A. Ashdown-Franks G. Hendrikse J. Sabiston C. M. Stubbs B. (2019). Physical activity and depression: towards understanding the antidepressant mechanisms of physical activity. Neurosci. Biobehav. Rev. 107, 525–539. doi: 10.1016/j.neubiorev.2019.09.040, 31586447

[ref22] Kraemer W. J. Ratamess N. A. French D. N. (2002). Resistance training for health and performance. Curr. Sports Med. Rep. 1, 165–171. doi: 10.1249/00149619-200206000-00007, 12831709

[ref23] Kreher J. B. Schwartz J. B. (2012). Overtraining syndrome: a practical guide. Sports Health 4, 128–138. doi: 10.1177/1941738111434406, 23016079 PMC3435910

[ref24] Li J. Liu F. Liu Z. Li M. Wang Y. Shang Y. . (2024). Prevalence and associated factors of depression in postmenopausal women: a systematic review and meta-analysis. BMC Psychiatry 24:431. doi: 10.1186/s12888-024-05875-0, 38858633 PMC11165857

[ref25] MacKenzie M. B. Kocovski N. L. (2016). Mindfulness-based cognitive therapy for depression: trends and developments. Psychol. Res. Behav. Manag. 9, 125–132. doi: 10.2147/PRBM.S63949, 27274325 PMC4876939

[ref26] Mawdsley D. Bennetts M. Dias S. Boucher M. Welton N. J. (2016). Model-based network Meta-analysis: a framework for evidence synthesis of clinical trial data. CPT Pharmacometrics Syst. Pharmacol. 5, 393–401. doi: 10.1002/psp4.12091, 27479782 PMC4999602

[ref27] Noetel M. Sanders T. Gallardo-Gómez D. Taylor P. Del Pozo Cruz B. van den Hoek D. . (2024). Effect of exercise for depression: systematic review and network meta-analysis of randomised controlled trials. BMJ 384:e075847. doi: 10.1136/bmj-2023-075847, 38355154 PMC10870815

[ref28] Pascoe M. C. Thompson D. R. Ski C. F. (2017). Yoga, mindfulness-based stress reduction and stress-related physiological measures: a meta-analysis. Psychoneuroendocrinology 86, 152–168. doi: 10.1016/j.psyneuen.2017.08.008, 28963884

[ref29] Pearce M. Garcia L. Abbas A. Strain T. Schuch F. B. Golubic R. . (2022). Association between physical activity and risk of depression: a systematic review and meta-analysis. JAMA Psychiatr. 79, 550–559. doi: 10.1001/jamapsychiatry.2022.0609, 35416941 PMC9008579

[ref30] Pérez-López F. R. Martínez-Domínguez S. J. Lajusticia H. Chedraui P. Health Outcomes Systematic Analyses Project (2017). Effects of programmed exercise on depressive symptoms in midlife and older women: a meta-analysis of randomized controlled trials. Maturitas 106, 38–47. doi: 10.1016/j.maturitas.2017.09.001, 29150165

[ref31] Piercy K. L. Troiano R. P. Ballard R. M. Carlson S. A. Fulton J. E. Galuska D. A. . (2018). The physical activity guidelines for Americans. JAMA 320, 2020–2028. doi: 10.1001/jama.2018.14854, 30418471 PMC9582631

[ref32] Rücker G. Schwarzer G. (2015). Ranking treatments in frequentist network meta-analysis works without resampling methods. BMC Med. Res. Methodol. 15:58. doi: 10.1186/s12874-015-0060-8, 26227148 PMC4521472

[ref33] Saeed S. A. Cunningham K. Bloch R. M. (2019). Depression and anxiety disorders: benefits of exercise, yoga, and meditation. Am. Fam. Physician 99, 620–627. 31083878

[ref34] Schuch F. B. Stubbs B. (2019). The role of exercise in preventing and treating depression. Curr. Sports Med. Rep. 18, 299–304. doi: 10.1249/JSR.0000000000000620, 31389872

[ref35] Schuch F. B. Vancampfort D. Firth J. Rosenbaum S. Ward P. B. Silva E. S. . (2018). Physical activity and incident depression: a Meta-analysis of prospective cohort studies. Am. J. Psychiatry 175, 631–648. doi: 10.1176/appi.ajp.2018.17111194, 29690792

[ref9003] Sen E. I. Esmaeilzadeh S. Eskiyurt N. (2020). Effects of whole-body vibration and high impact exercises on the bone metabolism and functional mobility in postmenopausal women. J Bone Miner Metab 38, 392–404. doi: 10.1007/s00774-019-01072-2 31897748

[ref36] Singh B. Olds T. Curtis R. Dumuid D. Virgara R. Watson A. . (2023). Effectiveness of physical activity interventions for improving depression, anxiety and distress: an overview of systematic reviews. Br. J. Sports Med. 57, 1203–1209. doi: 10.1136/bjsports-2022-106195, 36796860 PMC10579187

[ref37] Sterne J. A. C. Savović J. Page M. J. Elbers R. G. Blencowe N. S. Boutron I. . (2019). RoB 2: a revised tool for assessing risk of bias in randomised trials. BMJ 366:l4898. doi: 10.1136/bmj.l4898, 31462531

[ref38] Ter Veer E. van Oijen M. G. H. van Laarhoven H. W. M. (2019). The use of (network) Meta-analysis in clinical oncology. Front. Oncol. 9:822. doi: 10.3389/fonc.2019.00822, 31508373 PMC6718703

[ref39] Tian S. Liang Z. Qui F. Yu Y. Wang C. Zhang M. . (2024). Optimal exercise modality and dose to improve depressive symptoms in adults with major depressive disorder: a systematic review and Bayesian model-based network meta-analysis of RCTs. J. Psychiatr. Res. 176, 384–392. doi: 10.1016/j.jpsychires.2024.06.031, 38944017

[ref40] Wang H. Li S. Zhang X. Zhu Y. Huang Q. Guo K.-L. . (2025). Effects of different physical activity interventions on depressive symptoms in menopausal women: a systematic review and network meta-analysis. BMC Public Health 25:3088. doi: 10.1186/s12889-025-24398-1, 40993622 PMC12462009

[ref41] Wheeler D. C. Hickson D. A. Waller L. A. (2010). Assessing local model adequacy in Bayesian hierarchical models using the partitioned deviance information criterion. Comput Stat Data Anal 54, 1657–1671. doi: 10.1016/j.csda.2010.01.025, 21243121 PMC3020089

[ref42] World Health Organization (2020). WHO guidelines on physical activity and sedentary behaviour. Available at: https://www.who.int/publications/i/item/9789240015128 (Accessed November 9, 2025).

[ref43] World Health Organization (2025a). Depression. Available online at: https://www.who.int/health-topics/depression (Accessed November 9, 2025).

[ref44] World Health Organization (2025b). Depressive disorder (depression). Available online at: https://www.who.int/news-room/fact-sheets/detail/depression (Accessed November 9, 2025).

[ref45] Xu H. Liu J. Li P. Liang Y. (2024). Effects of mind-body exercise on perimenopausal and postmenopausal women: a systematic review and meta-analysis. Menopause 31, 457–467. doi: 10.1097/GME.0000000000002336, 38669625 PMC11465887

[ref46] Zou L. Yeung A. Li C. Wei G.-X. Chen K. W. Kinser P. A. . (2018). Effects of meditative movements on major depressive disorder: a systematic review and Meta-analysis of randomized controlled trials. J. Clin. Med. 7:195. doi: 10.3390/jcm7080195, 30071662 PMC6111244

