# Supplementary material for: Optimal exercise modality and dose for alleviating depressive symptoms in postmenopausal women: a systematic review and network meta-analysis of randomized controlled trials
Source: Front Psychol. 2025 Dec 11;16:1743949. doi: 10.3389/fpsyg.2025.1743949 (PMC12739755; doi:10.3389/fpsyg.2025.1743949)
Supplement: Supplementary file 1 [file Supplementary_file_1.docx]

**Optimal Exercise Modality and Dose for Alleviating Depressive Symptoms in Postmenopausal Women: A Systematic Review and Network Meta-Analysis of Randomized Controlled Trials**

**Supplemental Appendix**

**Catalog**

**Part 1: Systematic Review Methodology 1**

**Appendix 1.** Characteristics of the included studies **1**

**Appendix 2.** Search strategy **6**

**Appendix 3.** Risk of bias assessment **8**

**Part 2: Results of Network Meta-Analysis 10**

**Appendix 4.** Assessment of inconsistency within comparisons**. 10**

**Appendix 5.** Comparison-adjusted funnel plot of all studies **11**

**Appendix 6.** Rankograms, P-score plot, for the effects of different exercise interventions **12**

**Appendix 7.** Evaluation of the Certainty of evidence **13**

**Part 3: Results of Dose-Response Network Meta-Analyses 15**

**Appendix 8.** Dose-response network meta-analysis study characteristics **15**

**Appendix 9.** Three key assumptions of dose-response network meta-analysis **19**

**Appendix 10.** Models’ selection **22**

**Appendix 11.** Dose-response relationships **26**

**Appendix 12.** Ranking of effectiveness of different exercise dose **27**

**Appendix 13.** Sensitivity analysis and network meta-regression **28**

**Appendix 14.** Included studies **30**

**Part 1: Systematic Review Methodology**

**Appendix 1.** Characteristics of the included studies

| Author (year) | Country | Group | Sample size | Age (SD) | Intervention Program | Duration | Frequency | Length |
| --- | --- | --- | --- | --- | --- | --- | --- | --- |
| Abedi 2015 | Iran | EG | 53 | 52.4±3.8 | pedometer-based walking | NR | NR | 12 wk |
|  |  | CG | 53 | 53±4.1 | usual care |  |  |  |
| Abdoshahi 2023 | Iran | EG | 16 | 50–55 | Mind-body exercise | NR | 2 days/wk | 12 wk |
|  |  | CG | 16 |  | usual care |  |  |  |
| Afonso 2012 | Iran | EG2 | 24 | 50-65 | yoga | 1 hour/day | 2 days/wk | 16 wk |
|  |  | CG | 16 | 50-65 | usual care |  |  |  |
| Aibar 2019 | Spain | EG | 55 | 69.98±7.83 | Pilates-based exercise | 1 hour/day | 2 days/wk | 12 wk |
|  |  | CG | 55 | 66.79±10.14 | usual care |  |  |  |
| Bernard 2015 | France | EG | 61 | 65.46±4.37 | supervised and home-based walking | 40 minutes/day | 3 days/wk | 24 wk |
|  |  | CG | 60 | 65.5±4.03 | usual care |  |  |  |
| Blumenthal 1991 | USA | EG1 | 16 | 67.7±5.1 | aerobic exercise | 1 hour/day | 3 days/wk | 16 wk |
|  |  | EG2 | 17 | 67.7±5.1 | yoga |  | 2 days/wk |  |
|  |  | CG | 18 | 67.7±5.1 | usual care |  |  |  |
| Bowen 2006 | USA | EG | 86 | 50-75 | home-based aerobic exercise | 45 minutes/day | 5 days/wk | 48 wk |
|  |  | CG | 87 | 50-75 | stretching training |  |  |  |
| Carcelen 2022 | Spain | EG | 63 | 69.70±6.15 | Qigong | 1 hour/day | 2 days/wk | 12 wk |
|  |  | CG | 62 | 69.75±6.76 | usual care |  |  |  |
| Curi 2018 | Brazil | EG | 33 | 64.25±0.14 | supervised Pilates | 1 hour/day | 2 days/wk | 16 wk |
|  |  | CG | 31 | 63.75±0.08 | usual care |  |  |  |
| Elavsky 2007 | American | EG1 | 63 | 42–58 | Aerobic exercise | 1 hour/day | 3 days/wk | 16 wk |
|  |  | CG2 | 62 |  | Mind-body exercise | 90 min/day | 2 days/wk | 16 wk |
|  |  | EG | 39 |  | usual care |  |  |  |
| Elsayed 2022 | Egypt | EG | 30 | 58.79 ± 2.81 | Aerobic exercise | 30 min/day | 3 days/wk | 16 wk |
|  |  | CG | 30 | 58.79 ± 2.81 | usual care |  |  |  |
| Farzane 2022 | Iran | EG | 21 | 61.47±1.55 | Pilates | 75 minutes/day | 3 days/wk | 8 wk |
|  |  | CG | 17 | 62.00±1.4 | usual care |  |  |  |
| Gao 2016 | China | EG | 26 | 40–62 | Square dance | 75 min/day | 5 days/wk | 12 wk |
|  |  | CG | 24 |  | usual care |  |  |  |
| Gary 2007 | Atlanta | EG | 13 | 68±12 | home-based walking | 30 min/day | 3 days/wk | 12 wk |
|  |  | CG | 10 | 68±12 | health education |  |  |  |
| Gusi 2008 | Spain | EG | 64 | 71±5 | supervised walking-based exercise | 50 minutes/day | 3 days/wk | 24 wk |
|  |  | CG | 63 | 74±6 | routine care and a recommendation of physical activity |  |  |  |
| Gutierrez 2012 | Spain | EG | 30 | 60-70 | multicomponent exercise | 50-60 minutes/day | 3 day/wk | 24 wk |
|  |  | CG | 30 | 60-70 | usual care |  |  |  |
| Hu 2017 | China | EG | 46 | 52.60±4.12 | supervised walking | 1 hour/day | 3 days/wk | 16 wk |
|  |  | CG | 45 | 54.15±2.32 | usual care |  |  |  |
| Imayama 2011 | USA | EG | 117 | 58.1±5.0 | supervised aerobic exercise | 45 minutes | 5 days/wk | 48 wk |
|  |  | CG | 87 | 57.4±4.4 | usual care |  |  |  |
| Innes 2012 | USA | EG | 10 | 58.40±6.32 | yoga | 90 minutes/day | 2 days/wk | 8 wk |
|  |  | CG | 10 | 58.90±9.11 | educational film |  |  |  |
| Kabasakal 2025 | Turkey | EG | 13 | 59.45 ± 11.52 | Mind-body exercise | 1 hour/day | 2 days/wk | 6 wk |
|  |  | CG | 13 | 59.45 ± 11.52 | usual care |  |  |  |
| Kim 2019 | Korea | EG | 12 | 76.10±3.85 | strength training | 50-80  minutes/day | 3 days/wk | 24 wk |
|  |  | CG | 13 | 76.40±3.27 | usual care |  |  |  |
| Liu 2016 | China | EG | 32 | 66.3±2.7 | Tai Chi | 1 hour/day | 5 days/wk | 16 wk |
|  |  | CG | 32 | 65.8±3.2 | usual care |  |  |  |
| Luoto 2012 | Finland | EG | 88 | 54.5 ± 3.8 | Aerobic exercise | 50 minutes/day | 4 days/wk | 24 wk |
|  |  | CG | 88 | 54.2 ± 3.7 | usual care |  |  |  |
| Ma 2016 | China | EG | 40 | 60.9±5.1 | Qigong | 1 hour/day | 3 days/wk | 20 wk |
|  |  | CG | 40 | 60.2±4.1 | usual care |  |  |  |
| Newton 2015 | American | EG | 107 | 40–62 | Yoga | 90 min/day | 2 days/wk | 12 wk |
|  |  | CG | 142 |  | usual care |  |  |  |
| Noh 2020 | Korea | EG | 21 | 50–65 | Aerobic exercise | 1 hour/day | 3 days/wk | 12 wk |
|  |  | CG | 19 |  | Routine medical |  |  |  |
| Pang 2021 | Korea | EG | 13 | 60.89 ± 6.62 | Aerobic exercise | 1 hour/day | 5 days/wk | 12 wk |
|  |  | CG | 16 | 59.33 ± 6.54 | usual care |  |  |  |
| Pinheiro 2020 | Brazil | EG | 20 | 79±7.66 | supervised progressive resistance training | NR | 2 days/wk | 12 wk |
|  |  | CG | 20 | 81.7±5.95 | usual care |  |  |  |
| Shahidi 2011 | Iran | EG1 | 23 | 65.5±4.8 | laughter yoga | 30 min/day | NR | NR |
|  |  | EG2 | 23 | 65.7±4.2 | xercise therapy |  |  |  |
|  |  | CG | 24 | 68.4±6.3 | usual car |  |  |  |
| Sen 2019 | Turkey | EG | 19 | 53.1±4.4 | supervised aerobic, resistance and stretching exercise | 20-60 min/day | 3 days/wk | 24 wk |
|  |  | CG | 20 | 54.5±6.0 | usual care |  |  |  |
| Song 2022 | China | EG | 20 | 64.15±8.56 | Tai Chi | 1 hour/day | 3 days/wk | 12 wk |
|  |  | CG | 20 | 64.15±8.56 | wellness education |  |  |  |
| Soori 2022 | Iran | EG1 | 25 | 62.48±2.87 | aerobic exercise | 40 minutes/day | 3 days/wk | 3 wk |
|  |  | EG2 | 25 | 62.66±1.68 | Pilates |  |  |  |
|  |  | CG | 25 | 63.80±3.35 | usual care |  |  |  |
| Williams 1997 | Malaysia | EG | 94 | 71.8±5.6 | aerobic exercises and strengthening exercises | 1 hour/day | 2 days/wk | 42wk |
|  |  | CG | 93 | 71.6±5.2 | usual care |  |  |  |

**Appendix 2.** Search strategy

| **Database** | **Search strategy** | **Results** |
| --- | --- | --- |
| PubMed | ("postmenopause"[mh] OR "post menopausal period"[tiab] OR postmenopausal[tiab] OR "Post Menopause"[tiab] OR "Senior woman"[tiab] OR "Elderly woman"[tiab] OR "Older woman"[tiab]) AND ("exercise"[mh] OR exercises[tiab] OR "physical activity"[tiab] OR "aerobic exercise"[tiab] OR sport*[tiab] OR walk*[tiab] OR swim*[tiab] OR yoga[tiab] OR qigong[tiab] OR baduanjin[tiab] OR pilate[tiab] OR taichi[tiab] OR resistance[tiab] OR training[tiab]) AND ("depression"[mh] OR depress*[tiab]) AND ("randomized controlled trial"[pt] OR "controlled clinical trial"[pt] OR "randomized controlled trials as topic"[mh] OR "clinical trials as topic"[mh] OR "controlled clinical trials as topic"[mh] OR "clinical trial"[pt] OR "random allocation"[mh] OR randomized[tiab] OR randomised[tiab] OR randomization[tiab] OR randomly allocated[tiab] OR RCT[tiab] OR clinical trial*[tiab] OR clinical stud*[tiab]) | 77 |
| Web of Science | TS=(("postmenopause" OR "post menopausal period" OR postmenopausal OR "Post Menopause" OR "Senior woman" OR "Elderly woman" OR "Older woman") AND ("exercise" OR exercises OR "physical activity" OR "aerobic exercise" OR sport* OR walk* OR swim* OR yoga OR qigong OR baduanjin OR pilate OR taichi OR resistance OR training) AND (depression OR depressive) AND ("randomized controlled trial" OR "controlled clinical trial" OR "clinical trial" OR "random allocation" OR randomized OR randomised OR randomization OR RCT)) NOT DT=("Review Article" OR "Review") | 98 |
| Cochrane | #1 [mh postmenopause] #2 (postmenopause OR postmenopausal OR "post menopausal" OR "Senior woman" OR "Elderly woman" OR "Older woman"):ti,ab,kw #3 [mh exercise] #4 (exercise* OR "physical activity" OR "aerobic exercise" OR sport* OR walk* OR swim* OR yoga OR qigong OR baduanjin OR pilate OR taichi OR resistance OR training):ti,ab,kw #5 [mh depression] #6 (depression OR depressive):ti,ab,kw #7 #1 OR #2 #8 #3 OR #4 #9 #5 OR #6 #10 #7 AND #8 AND #9 | 786 |
| EBSCO | ( (MH "Postmenopause+") OR TI("postmenopause" OR "post menopausal period" OR postmenopausal OR "Post Menopause" OR "Senior woman" OR "Elderly woman" OR "Older woman") OR AB("postmenopause" OR "post menopausal period" OR postmenopausal OR "Post Menopause" OR "Senior woman" OR "Elderly woman" OR "Older woman") ) AND ( (MH "Exercise+") OR TI("exercise" OR exercises OR "physical activity" OR "aerobic exercise" OR sport* OR walk* OR swim* OR yoga OR qigong OR baduanjin OR pilate OR taichi OR resistance OR training) OR AB("exercise" OR exercises OR "physical activity" OR "aerobic exercise" OR sport* OR walk* OR swim* OR yoga OR qigong OR baduanjin OR pilate OR taichi OR resistance OR training) ) AND ( (MH "Depression+") OR TI(depression OR depressive) OR AB(depression OR depressive) ) AND ( (MH "Randomized Controlled Trials+") OR PT "Randomized Controlled Trial" OR TI("randomized controlled trial" OR "controlled clinical trial" OR "clinical trial" OR "random allocation" OR randomized OR randomised OR randomization OR RCT) OR AB("randomized controlled trial" OR "controlled clinical trial" OR "clinical trial" OR "random allocation" OR randomized OR randomised OR randomization OR RCT) ) | 166 |
| Embase | ('postmenopause'/exp OR 'post menopausal':ab,ti OR postmenopausal:ab,ti OR 'Senior woman':ab,ti OR 'Elderly woman':ab,ti OR 'Older woman':ab,ti) AND ('exercise'/exp OR exercises:ab,ti OR 'physical activity':ab,ti OR sport*:ab,ti OR 'aerobic exercise':ab,ti OR walk*:ab,ti OR qigong:ab,ti OR swim*:ab,ti OR yoga:ab,ti OR baduanjin:ab,ti OR pilate:ab,ti OR taichi:ab,ti OR resistance:ab,ti OR training:ab,ti) AND ('depression'/exp OR depress*:ab,ti) AND ('randomized controlled trial'/exp OR randomized:ab,ti OR randomised:ab,ti OR randomization:ab,ti OR randomly:ab,ti OR RCT:ab,ti OR 'controlled clinical trial'/exp OR 'controlled clinical':ab,ti) | 112 |

**Appendix 3.** Risk of bias assessment.

| **Study** | **Random sequence**  **generation** | **Deviations from intended interven** | **Missing outcome data** | **Measurement of the outcome** | **Seleetion of the reported result** | **Risk category** |
| --- | --- | --- | --- | --- | --- | --- |
| Abedi 2015 | ? | + | + | + | + | Moderate |
| Abdoshahi 2023 | + | - | + | + | + | High |
| Afonso 2012 | ? | + | - | + | + | High |
| Aibar 2019 | + | + | + | + | + | Low |
| Bernard 2015 | + | + | + | + | + | Low |
| Blumenthal 1991 | ? | + | + | + | + | Moderate |
| Bowen 2006 | ? | + | + | + | + | Moderate |
| Carcelen 2022 | + | + | + | + | + | Low |
| Curi 2018 | ? | ? | + | + | + | Moderate |
| Elavsky 2007 | + | ? | + | + | + | Moderate |
| Elsayed 2022 | + | ? | + | + | + | Moderate |
| Farzane 2022 | + | + | + | + | + | Low |
| Gao 2016 | + | ? | + | + | + | Moderate |
| Gary 2007 | - | ? | + | **?** | + | High |
| Gusi 2008 | + | ? | + | + | + | Moderate |
| Gutierrez 2012 | **?** | ? | + | + | **?** | Moderate |
| Hu 2017 | + | ? | + | + | + | Moderate |
| Imayama 2011 | + | + | + | + | + | Low |
| Innes 2012 | + | + | + | **?** | **?** | Moderate |
| Kabasakal | **?** | **?** | + | + | + | Moderate |
| Kim 2019 | + | **?** | + | + | + | Moderate |
| Liu 2016 | **?** | **?** | + | + | + | Moderate |
| Luoto 2012 | **?** | **?** | + | + | + | Moderate |
| Ma 2016 | **?** | **?** | + | **?** | + | Moderate |
| Newton 2015 | ? | ? | + | + | + | Moderate |
| Noh 2020 | ? | ? | + | + | + | Moderate |
| Pang 2021 | + | ? | + | + | + | Moderate |
| Pinheiro 2020 | + | ? | ? | + | + | Moderate |
| Shahidi 2011 | ? | ? | + | ? | + | Moderate |
| Sen 2019 | ? | + | + | ? | + | Moderate |
| Song 2022 | + | + | + | + | + | Low |
| Soori 2022 | + | ? | + | + | + | Moderate |
| Williams 1997 | ? | ? | + | ? | + | Moderate |

**Part 2: Results of Network Meta-Analysis**

**Appendix 4.** Assessment of inconsistency within comparisons.

**Table 4-1.** Assessment of inconsistency within comparisons

| **Comparison** | **k** | **Direct** | **Indirect** | **Difference** | **z** | **P-value** |
| --- | --- | --- | --- | --- | --- | --- |
| AE vs CBE | NA | NA | -0.04 | NA | NA | NA |
| AE vs MBE | 4 | -0.06 | -0.09 | 0.030 | 0.07 | 0.946 |
| AE vs RE | NA | NA | 0.23 | NA | NA | NA |
| AE vs CON | 18 | 0.64 | 1.48 | -0.84 | -1.29 | 0.199 |
| CBE vs MBE | NA | NA | 0.12 | NA | NA | NA |
| CBE vs RE | NA | NA | 0.27 | NA | NA | NA |
| CBE vs CON | 4 | 0.60 | NA | NA | -5.17 | 0.001 |
| MBE vs RE | NA | NA | 0.15 | NA | NA | NA |
| MBE vs CON | 12 | 0.70 | 1.31 | -0.61 | -1.02 | 0.308 |
| RE vs CON | 3 | -0.85 | NA | NA | NA | NA |

***Note:*** AE: Aerobic exercise, CBE: Combined exercise, MBE: Mind-body exercise, RE: Resistance exercise; CON: Control group


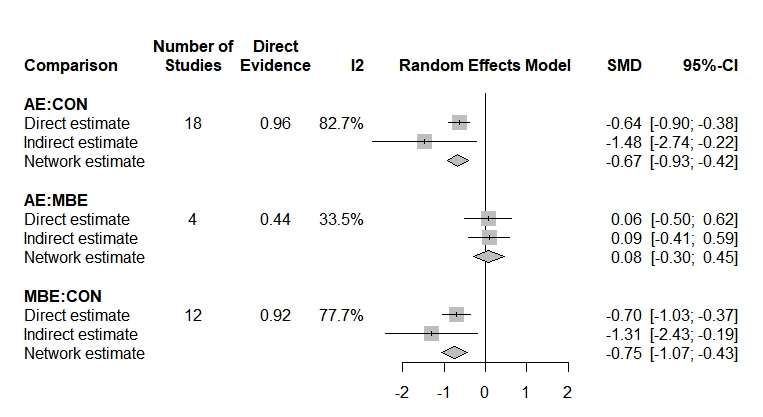


**Figure 4-1. Dot splitting method to explore inconsistency**. AE: Aerobic exercise, MBE: Mind-body exercise, CON: Control group.

**Appendix 5.** Comparison-adjusted funnel plot of all studies.


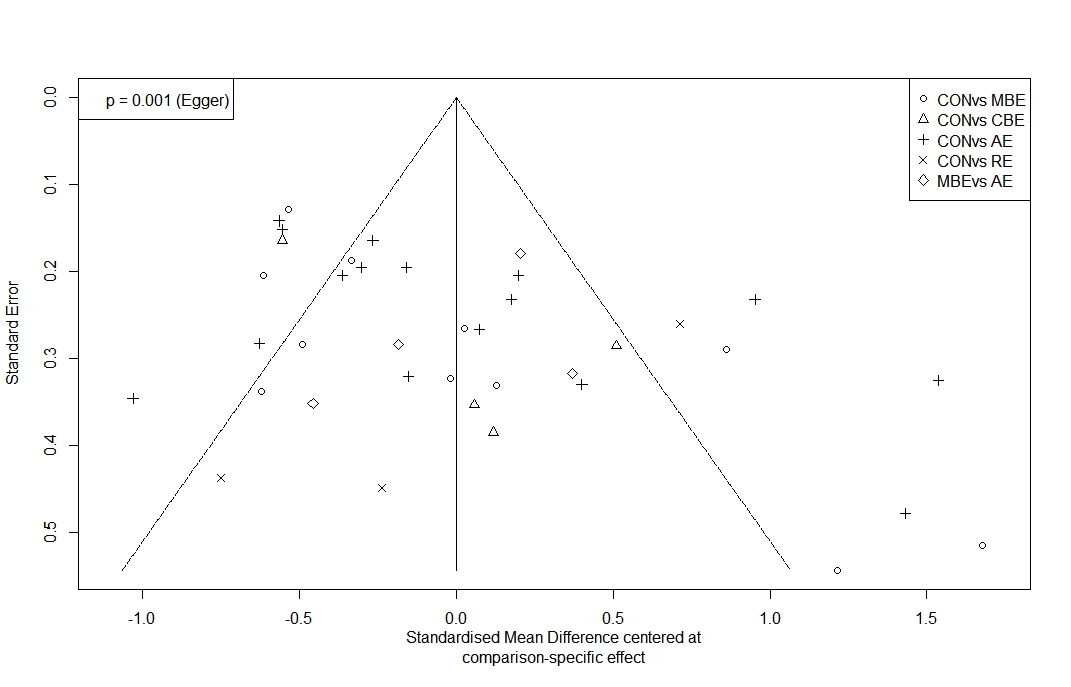


**Figure 5-1. Comparison-adjusted funnel plot of all studies**. AE: Aerobic exercise, MBE: Mind-body exercise, CON: Control group.


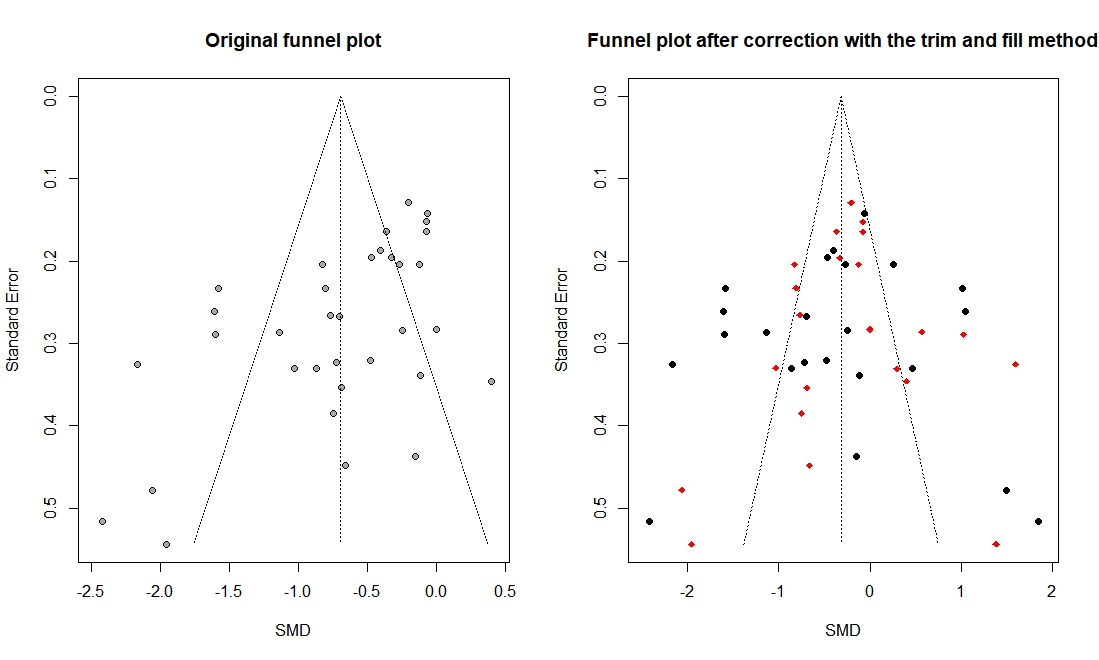


**Figure 5-2.** Original funnel plot and funnel plot corrected by the trim and fill method.

**Appendix 6.** Rankograms, P-score plot, for the effects of different exercise interventions


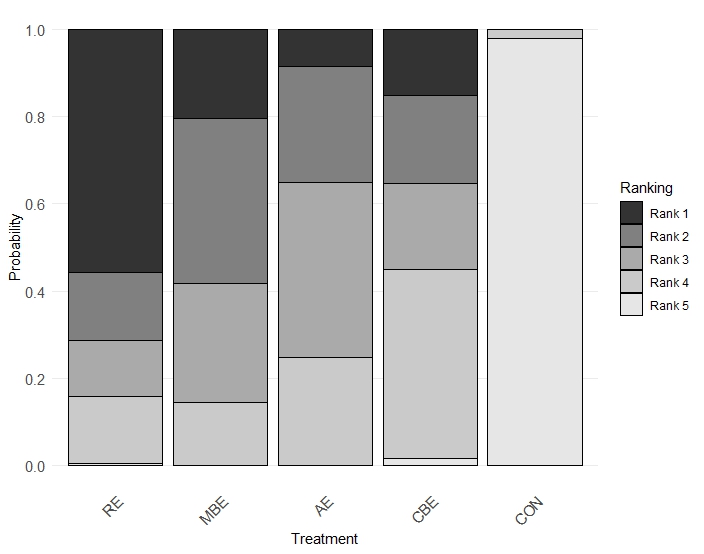


**Figure 6-1. Rrank probability plot**

**
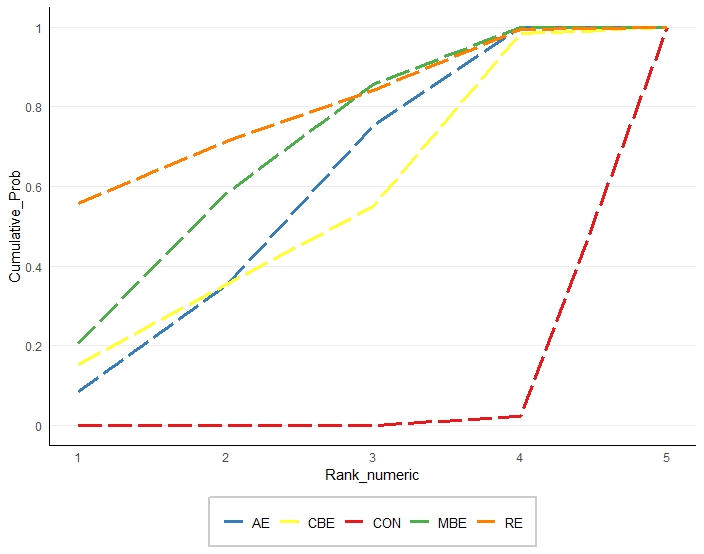
**

**Figure 6-2. P-score plot**

**Appendix 7.** Evaluation of the Certainty of evidence

| Comparison | N | Within-study bias | Reporting bias | Indirectness | Imprecision | Heterogeneity | Incoherence | Confidence | Reasons |
| --- | --- | --- | --- | --- | --- | --- | --- | --- | --- |
| Mixed evidence | | | | | | | | | |
| CON vs AE | 18 | Some concerns | Undetected | No concerns | No concerns | Major concerns | No concerns | Very low | -1^*^ Within-study bias  -2^*^ Heterogeneity |
| CON vs RE | 3 | Some concerns | Undetected | No concerns | Some concerns | Some concerns | No concerns | Very low | -1^*^ Within-study bias  -1^*^ Imprecision  -1^*^ Heterogeneity |
| CON vs MBE | 12 | Some concerns | Undetected | No concerns | No concerns | No concerns | No concerns | Moderate | -1^*^ Within-study bias |
| CON vs CBE | 4 | Some concerns | Undetected | No concerns | Some concerns | Some concerns | No concerns | Very low | -1^*^ Within-study bias  -1^*^ Imprecision  -1^*^ Heterogeneity |
| AE vs MBE | 4 | Some concerns | Undetected | No concerns | No concerns | Major concerns | No concerns | Very low | -1^*^ Within-study bias  -2^*^ Heterogeneity |
| Indirect evidence | | | | | | | | | |
| AE vs CBE | - | Some concerns | Undetected | No concerns | Major concerns | No concerns | No concerns | Very low | -1^*^ Within-study bias  -2^*^ Imprecision |
| AE vs RE | - | Some concerns | Undetected | No concerns | Some concerns | Some concerns | No concerns | Very low | -1^*^ Within-study bias  -1^*^ Imprecision  -1^*^ Heterogeneity |
| CBE vs MBE | - | Some concerns | Undetected | No concerns | Major concerns | No concerns | No concerns | Very low | -1^*^ Within-study bias  -2^*^ Imprecision |
| CBE vs RE | - | Some concerns | Undetected | No concerns | Major concerns | No concerns | No concerns | Very low | -1^*^ Within-study bias  -2^*^ Imprecision |
| MBE vs RE |  | Some concerns | Undetected | No concerns | Major concerns | No concerns | No concerns | Very low | -1^*^ Within-study bias  -2^*^ Imprecision |

***Note:*** AE: Aerobic exercise, CBE: Combined exercise, MBE: Mind-body exercise, RE: Resistance exercise; CON: Control group.

−1* signifies a one-level downgrading of each relative treatment effect’s confidence.

−2* signifies a two-level downgrading of each relative treatment effect’s confidence.

**Part 3: Results of Dose-Response Network Meta-Analyses**

**Appendix 8. Dose-response network meta-analysis study characteristics**

| **Study** | **N** | **Agent** | **Exact dose** | **Dose** | **Residual dose** | **Frequency** | **Min/day** |
| --- | --- | --- | --- | --- | --- | --- | --- |
| Afonso 2012 | 15 | MBE | 480 | 600 | 120 | 2 | 60 |
|  | 15 | CON | 0 | 0 | 0 | 0 | 0 |
| Aibar 2019 | 54 | AE | 196 | 300 | 104 | 2 | 35 |
|  | 50 | CON | 0 | 0 | 0 | 0 | 0 |
| Bernard 2015 | 53 | AE | 456 | 600 | 144 | 3 | 40 |
|  | 55 | CON | 0 | 0 | 0 | 0 | 0 |
| Blumenthal 1991 | 16 | AE | 918 | 900 | 18 | 3 | 45 |
|  | 17 | MBE | 276 | 300 | 24 | 2 | 60 |
|  | 18 | CON | 0 | 0 | 0 | 0 | 0 |
| Bowen 2006 | 86 | AE | 1642.5 | 1200 | 442.5 | 5 | 45 |
|  | 87 | CON | 0 | 0 | 0 | 0 | 0 |
| Carcelen 2022 | 57 | MBE | 330 | 300 | 30 | 2 | 50 |
|  | 60 | CON | 0 | 0 | 0 | 0 | 0 |
| Curi 2018 | 31 | MBE | 216 | 300 | 84 | 2 | 60 |
|  | 30 | CON | 0 | 0 | 0 | 0 | 0 |
| Elavsky 2007 | 63 | AE | 513 | 600 | 87 | 3 | 45 |
|  | 62 | MBE | 414 | 300 | 114 | 2 | 90 |
|  | 39 | CON | 0 | 0 | 0 | 0 | 0 |
| Elsayed 2022 | 30 | AE | 432 | 300 | 132 | 3 | 30 |
|  | 30 | CON | 0 | 0 | 0 | 0 | 0 |
| Farzane 2022 | 15 | AE | 675 | 600 | 75 | 3 | 75 |
|  | 12 | CON | 0 | 0 | 0 | 0 | 0 |
| Gao 2016 | 32 | AE | 1590 | 1200 | 390 | 5 | 60 |
|  | 28 | CON | 0 | 0 | 0 | 0 | 0 |
| Gary 2007 | 13 | AE | 270 | 300 | 30 | 3 | 30 |
|  | 10 | CON | 0 | 0 | 0 | 0 | 0 |
| Gusi 2008 | 55 | AE | 720 | 600 | 120 | 3 | 50 |
|  | 51 | CON | 0 | 0 | 0 | 0 | 0 |
| Gutierrez 2012 | 27 | CBE | 1095 | 1200 | 105 | 3 | 50 |
|  | 30 | CON | 0 | 0 | 0 | 0 | 0 |
| Hu 2017 | 40 | AE | 456 | 600 | 144 | 3 | 40 |
|  | 40 | CON | 0 | 0 | 0 | 0 | 0 |
| Imayama 2011 | 116 | AE | 1642.5 | 1200 | 442.5 | 5 | 45 |
|  | 87 | CON | 0 | 0 | 0 | 0 | 0 |
| Innes 2012 | 10 | MBE | 414 | 300 | 114 | 2 | 90 |
|  | 10 | CON | 0 | 0 | 0 | 0 | 0 |
| Kabasakal 2025 | 13 | MBE | 144 | 300 | 156 | 2 | 40 |
|  | 13 | CON | 0 | 0 | 0 | 0 | 0 |
| Kim 2019 | 11 | RE | 472.5 | 600 | 127.5 | 3 | 45 |
|  | 10 | CON | 0 | 0 | 0 | 0 | 0 |
| Liu 2016 | 32 | MBE | 825 | 900 | 75 | 5 | 50 |
|  | 31 | CON | 0 | 0 | 0 | 0 | 0 |
| Luoto 2012 | 74 | AE | 1460 | 1200 | 260 | 4 | 50 |
|  | 77 | CON | 0 | 0 | 0 | 0 | 0 |
| Ma 2016 | 38 | RE | 594 | 600 | 6 | 3 | 60 |
|  | 40 | CON | 0 | 0 | 0 | 0 | 0 |
| Newton 2015 | 140 | MBE | 414 | 300 | 114 | 2 | 90 |
|  | 21 | CON | 0 | 0 | 0 | 0 | 0 |
| Noh 2020 | 19 | AE | 864 | 900 | 36 | 3 | 60 |
|  | 18 | CON | 0 | 0 | 0 | 0 | 0 |
| Pang 2021 | 12 | CBE | 1140 | 1200 | 60 | 5 | 60 |
|  | 10 | CON | 0 | 0 | 0 | 0 | 0 |
| Pinheiro 2020 | 11 | RE | 280 | 300 | 20 | 2 | 40 |
|  | 20 | CON | 0 | 0 | 0 | 0 | 0 |
| Shahidi 2011 | 20 | AE | 360 | 300 | 60 | 2 | 30 |
|  | 20 | MBE | 138 | 300 | 162 | 2 | 30 |
|  | 16 | CON | 0 | 0 | 0 | 0 | 0 |
| Sen 2019 | 18 | CBE | 877.5 | 900 | 22.5 | 3 | 45 |
|  | 20 | CON | 0 | 0 | 0 | 0 | 0 |
| Song 2022 | 20 | MBE | 396 | 300 | 96 | 3 | 40 |
|  | 25 | CON | 0 | 0 | 0 | 0 | 0 |
| Soori 2022 | 25 | AE | 576 | 600 | 24 | 3 | 40 |
|  | 25 | MBE | 336 | 300 | 36 | 3 | 40 |
|  | 71 | CON | 0 | 0 | 0 | 0 | 0 |
| Williams 1997 | 78 | CBE | 660 | 600 | 60 | 2 | 60 |
|  | 15 | CON | 0 | 0 | 0 | 0 | 0 |

**Appendix 9.** Three key assumptions of dose-response network meta-analysis

**9.1 Network Connectivity**

**
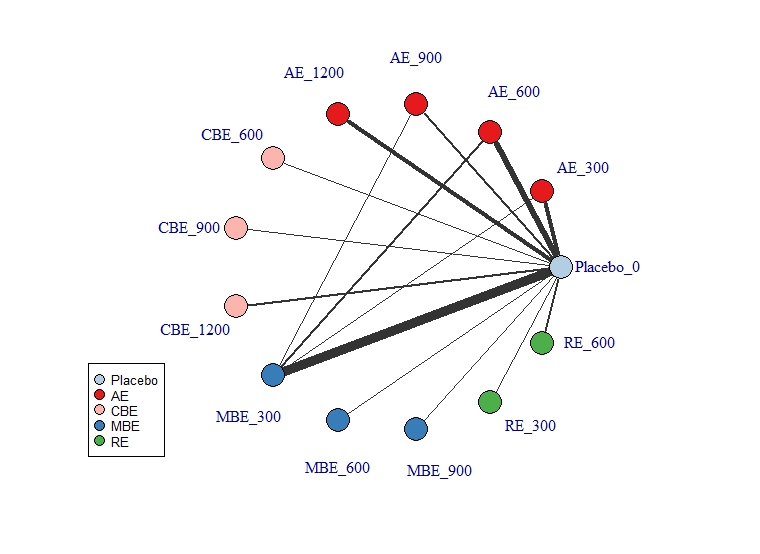
**

**Figure 8-1. Treatment-level network.**

**9.2 Network consistency**

**Table 9-1. Comparison of consistency and UME model fit**

| **Model** | **pD** | **Deviance** | **Residual deviance** | **DIC** |
| --- | --- | --- | --- | --- |
| Consistent | 41.9 | 103.5 | 52 | 146 |
| UME | 42.2 | 103.7 | 52.2 | 146.8 |

**9.3 Network transitivity**

**Table 9-2. Node-splitting analysis for consistency assessment**

| **Comparison** | **P-value** | **Median** | **95%CrI** | |
| --- | --- | --- | --- | --- |
| RE_600 vs Placebo_0 | 0.321 |  |  |  |
| -> direct |  | -0.489 | -1.486 | 0.541 |
| -> indirect |  | -0.417 | -6.014 | 5.264 |
| -> MBNMA |  | -0.465 | -1.485 | 0.543 |
| RE_300 vs Placebo_0 | 0.339 |  |  |  |
| -> direct |  | -0.186 | -3.052 | 2.624 |
| -> indirect |  | -0.234 | -0.79 | 0.271 |
| -> MBNMA |  | -0.233 | -0.742 | 0.271 |
| MBE_900 vs Placebo_0 | 0.503 |  |  |  |
| -> direct |  | -1.587 | -2.797 | -0.379 |
| -> indirect |  | -2.505 | -4.188 | -0.73 |
| -> MBNMA |  | -1.876 | -2.887 | -0.935 |
| MBE_600 vs Placebo_0 | 0.209 |  |  |  |
| -> direct |  | -0.209 | -6.386 | 5.649 |
| -> indirect |  | -1.259 | -1.925 | -0.613 |
| -> MBNMA |  | -1.25 | -1.925 | -0.624 |
| MBE_300 vs Placebo_0 | 0.481 |  |  |  |
| -> direct |  | -0.849 | -1.389 | -0.26 |
| -> indirect |  | -0.538 | -0.916 | -0.127 |
| -> MBNMA |  | -0.625 | -0.962 | -0.312 |
| CBE_1200 vs Placebo_0 | 0.572 |  |  |  |
| -> direct |  | -1.122 | -2.622 | 0.52 |
| -> indirect |  | -0.468 | -4.372 | 3.428 |
| -> MBNMA |  | -1.031 | -2.441 | 0.406 |
| CBE_900 vs Placebo_0 | 0.441 |  |  |  |
| -> direct |  | -0.673 | -4.544 | 3.397 |
| -> indirect |  | -0.763 | -1.898 | 0.338 |
| -> MBNMA |  | -0.773 | -1.831 | 0.305 |
| CBE_600 vs Placebo_0 | 0.451 |  |  |  |
| -> direct |  | -0.14 | -2.719 | 2.431 |
| -> indirect |  | -0.55 | -1.325 | 0.245 |
| -> MBNMA |  | -0.516 | -1.22 | 0.203 |
| AE_1200 vs Placebo_0 | 0.291 |  |  |  |
| -> direct |  | -0.558 | -1.184 | 0.152 |
| -> indirect |  | -1.492 | -2.679 | -0.317 |
| -> MBNMA |  | -0.742 | -1.351 | -0.203 |
| AE_900 vs Placebo_0 | 0.264 |  |  |  |
| -> direct |  | 0.079 | -2.798 | 3.188 |
| -> indirect |  | -0.579 | -1.053 | -0.156 |
| -> MBNMA |  | -0.556 | -1.013 | -0.152 |
| AE_600 vs Placebo_0 | 0.299 |  |  |  |
| -> direct |  | -0.781 | -1.508 | -0.143 |
| -> indirect |  | -0.301 | -0.636 | 0.021 |
| -> MBNMA |  | -0.371 | -0.675 | -0.101 |
| AE_300 vs Placebo_0 | 0.174 |  |  |  |
| -> direct |  | -0.747 | -1.847 | 0.284 |
| -> indirect |  | -0.179 | -0.34 | -0.037 |
| -> MBNMA |  | -0.185 | -0.338 | -0.051 |

**Appendix 10.** Models’ selection

**10.1 Non-linear functions and models fit comparison**

**
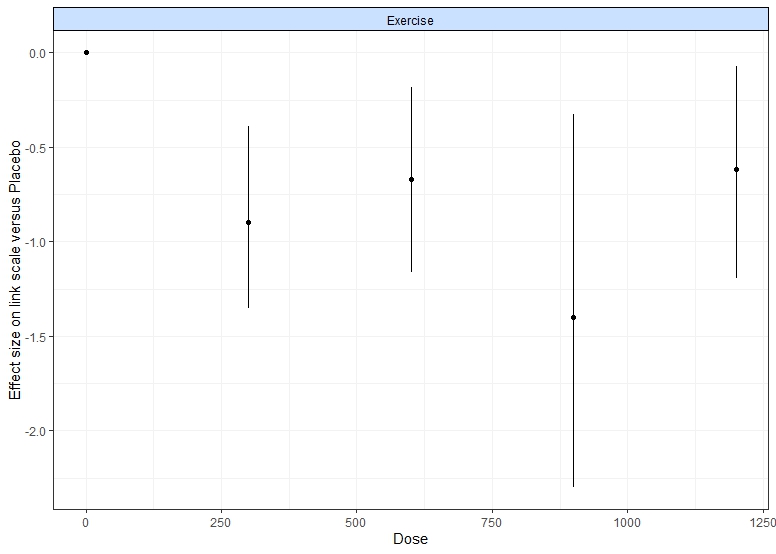
**

**Figure 10-1. “Split” NMA of overall exercise**

**
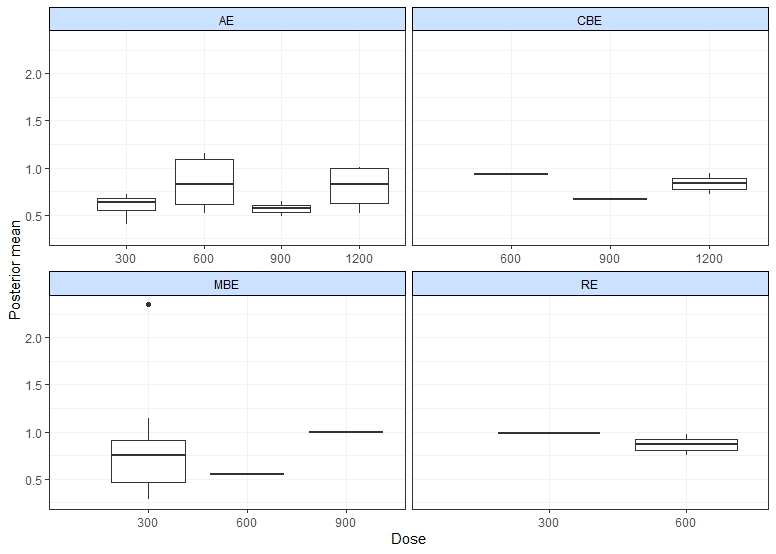
**

**Figure 10-2. “Split” NMA of different exercise**

**10.2 Models selection**

| **Model** | **DIC** | **SD** | **Deviance** | **Residual**  **deviance** | **pD** |
| --- | --- | --- | --- | --- | --- |
| Emax  (random treatment effects) | 214.3 | NA | 178.1 | 126.6 | 35.2 |
| Restricted cubic spline  (common treatment effects; 3 knots) | 227.3 | NA | 183.6 | 132.1 | 43 |
| Restricted cubic spline  (random treatment effects; 3 knots) | 158.1 | 0.47  (0.19, 0.93) | 107.6 | 56.1 | 50 |
| Exponential  (common treatment effects) | 153 | NA | 104.4 | 52.9 | 48 |
| Quadratic  (random treatment effects) | 151.6 | 0.44  (0.17,0.87) | 105 | 53.5 | 45.9 |

***Note:*** DIC = Deviance Information Criterion; SD = Between-study Standard Deviation; pD: Number of estimated parameters; NA = Not Applicable. The SD is presented as the main value and (95% Credible Intervals).

In addition to the model fit index, a deviation plot showing the contribution of each data point to the residuals can also help to confirm the robustness of the model selection. The contribution of each data point to the posterior mean bias should be around 1, which indicates a good model fit.


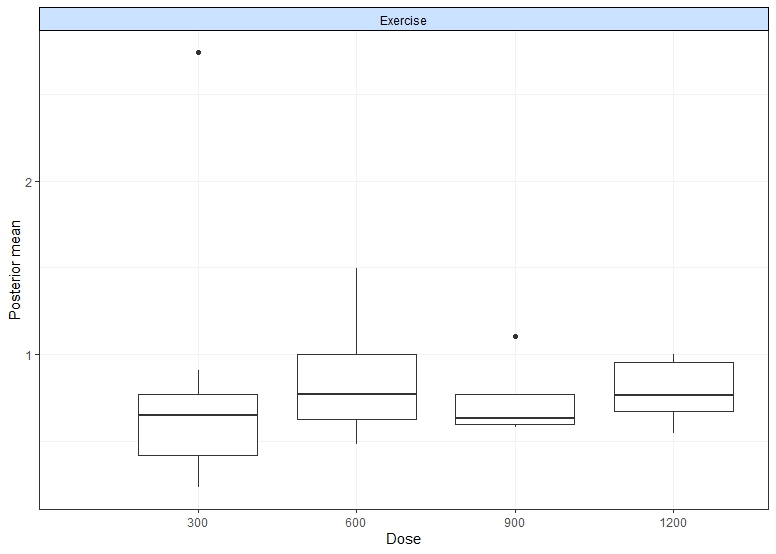


**Figure 10-3. Deviance plot at overall exercise level.**

**
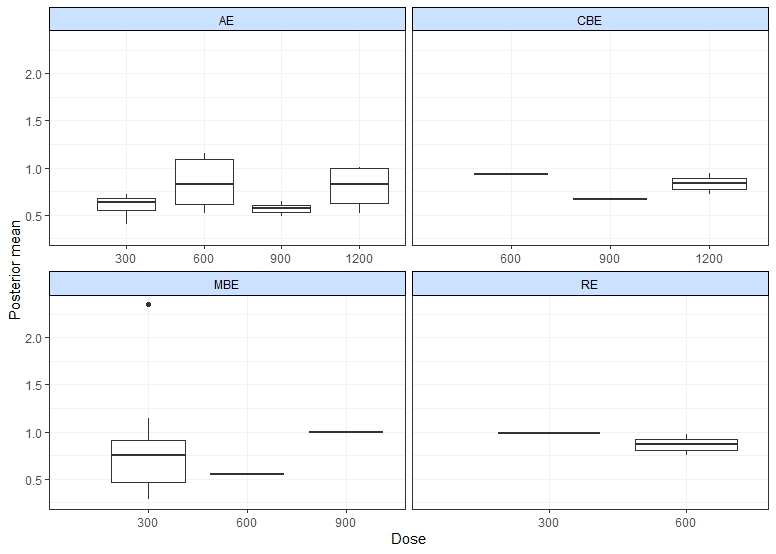
**

**Figure 10-4. Deviance plots at treatment-level.**

In order to determine the appropriateness of the model, we conducted a goodness-of-fit analysis. The goodness-of-fit metrics are displayed as interconnected lines, with the original dataset observations being represented by individual data points. These visuals are utilized to evaluate the model’s ability to accommodate variations in exercise and dosage-response functions

**
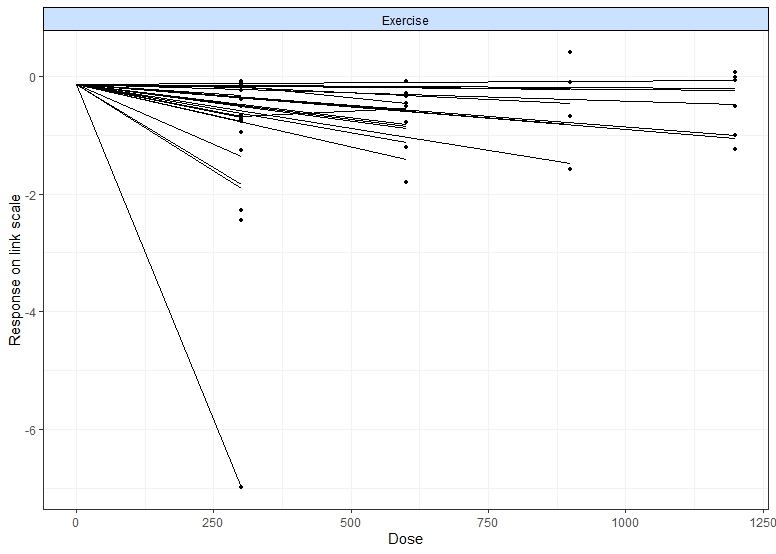
**

**Figure 10-5. Fit plots at overall exercise level.**

**
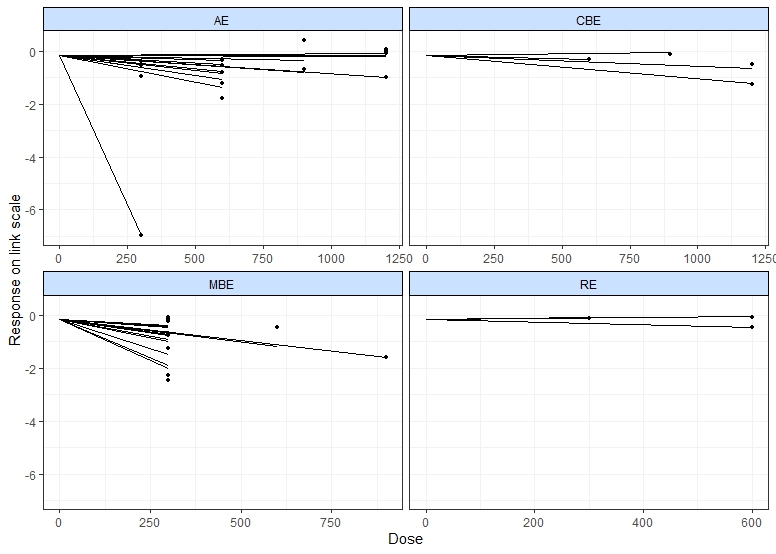
**

**Figure 10-6. Fit plots at agent-level.**

**Appendix 11.** Dose-response relationships

**
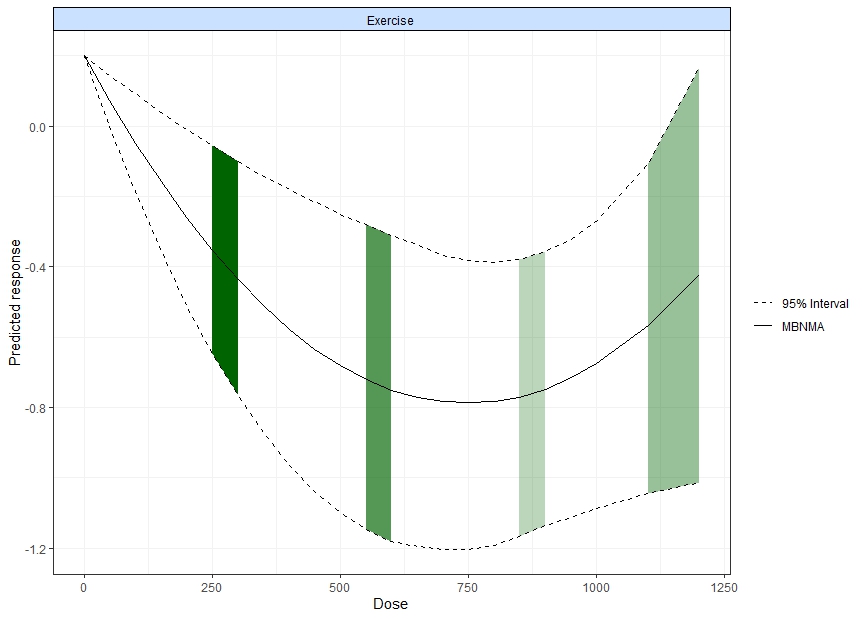
**

**Figure 11-1. Exercise dose-response relationship at agent-level.**

**
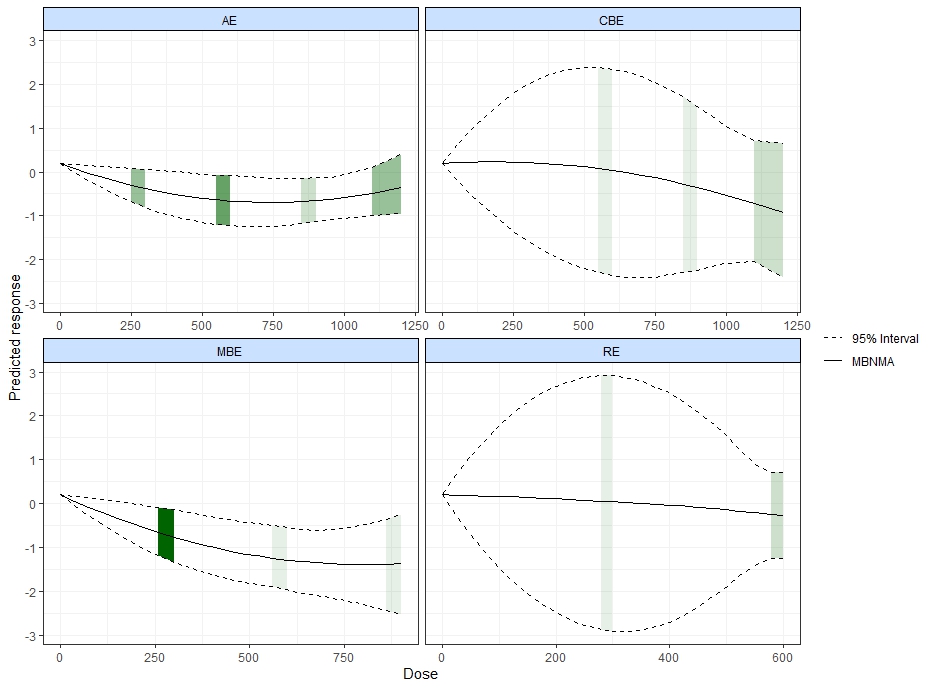
**

**Figure 11-2. Number of original studies at different dose scenarios**

the darker the color, the larger the amount of data.

**Appendix 12.** Ranking of effectiveness of different exercise dose


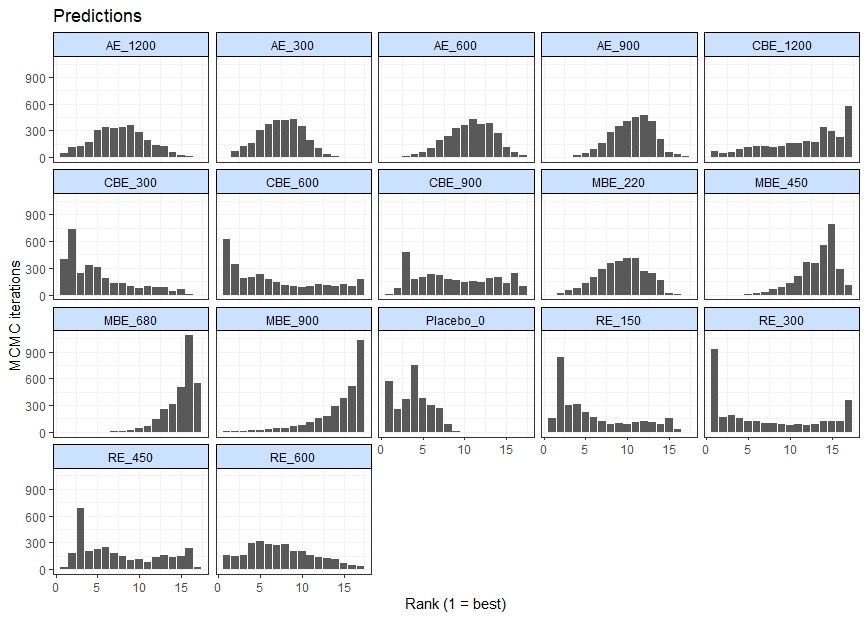


**Figure 12-1. Effectiveness ranking by different exercise dose**

**Appendix 13.** Sensitivity analysis and network meta-regression

**
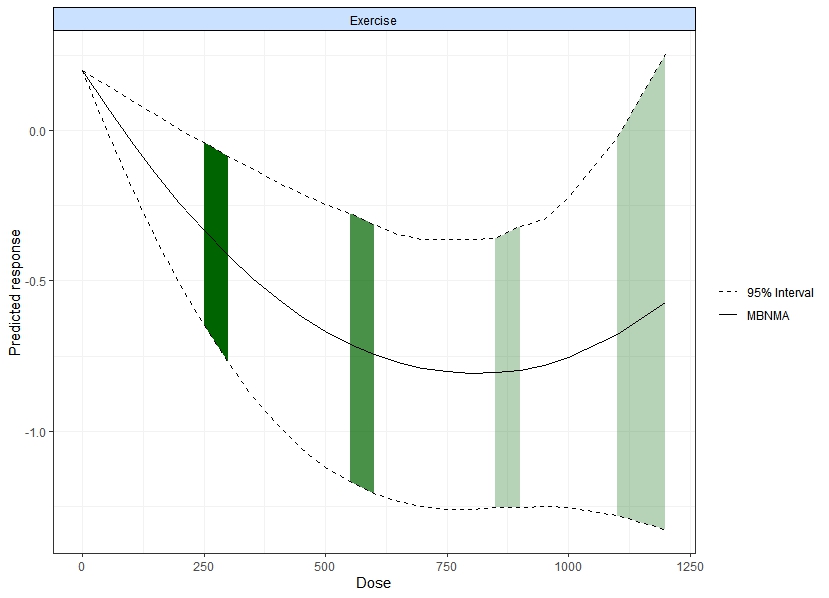
**

**Figure 13-1. Dose-response sensitivity analysis for overall exercise**

**
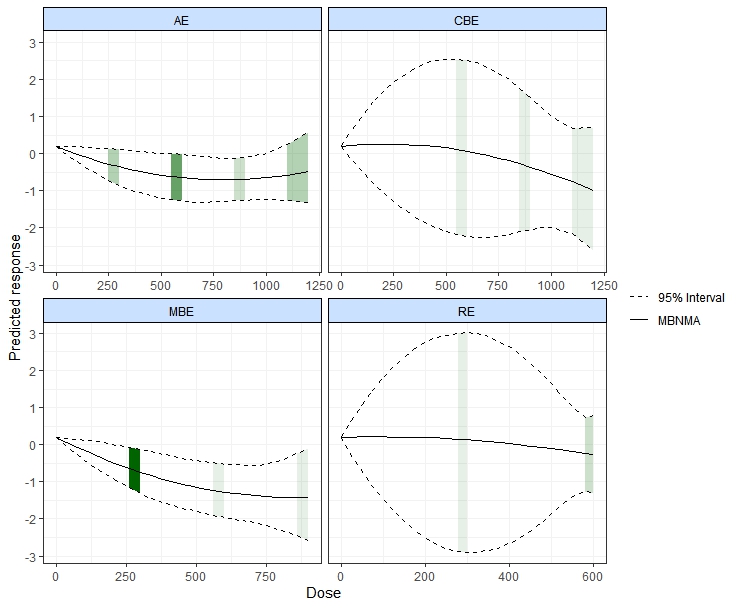
**

**Figure 13-2. Dose-response sensitivity analysis for different types of exercise**

**Table 13-1. Network Meta-Regression Results**

| **Moderator Variable** | **N** | **Coefficient** | **95% CI** | **SE** | **P-value** | **Residual Heterogeneity** | **τ²** |
| --- | --- | --- | --- | --- | --- | --- | --- |
| Supervision | 41 | 0.264 | [-0.420, 0.949] | 0.349 | 0.449 | QE = 189.25, P < .001 | 0.333 |
| Mean age | 41 | 0.0124 | [-0.0180, 0.0429] | 0.0155 | 0.424 | QE = 197.48, P < .001 | 0.337 |

**Appendix 14.** Included studies

Abdoshahi, M. (2023). The Impact of Pilates Training on Mental Health and Happiness among Untrained Menopausal Women. *Women’s Health Bulletin* 10, 96–103. doi: 10.30476/whb.2023.97578.1211

Abedi, P., Nikkhah, P., and Najar, S. (2015). Effect of pedometer-based walking on depression, anxiety and insomnia among postmenopausal women. *Climacteric* 18, 841–845. doi: 10.3109/13697137.2015.1065246

Afonso, R. F., Hachul, H., Kozasa, E. H., Oliveira, D. de S., Goto, V., Rodrigues, D., et al. (2012). Yoga decreases insomnia in postmenopausal women: a randomized clinical trial. *Menopause* 19, 186–193. doi: 10.1097/gme.0b013e318228225f

Aibar-Almazán, A., Hita-Contreras, F., Cruz-Díaz, D., de la Torre-Cruz, M., Jiménez-García, J. D., and Martínez-Amat, A. (2019). Effects of Pilates training on sleep quality, anxiety, depression and fatigue in postmenopausal women: A randomized controlled trial. *Maturitas* 124, 62–67. doi: 10.1016/j.maturitas.2019.03.019

Arslan Kabasakal, S. (2025). The effects of a 6-week pilates exercises on quality of life, depression, and musculoskeletal disorders in menopausal women. *The European Research Journal* 11, 296–303. doi: 10.18621/eurj.1603630

Bernard, P., Ninot, G., Bernard, P. L., Picot, M. C., Jaussent, A., Tallon, G., et al. (2015). Effects of a six-month walking intervention on depression in inactive post-menopausal women: a randomized controlled trial. *Aging Ment Health* 19, 485–492. doi: 10.1080/13607863.2014.948806

Blumenthal, J. A., Emery, C. F., Madden, D. J., Schniebolk, S., Walsh-Riddle, M., George, L. K., et al. (1991). Long-term effects of exercise on psychological functioning in older men and women. *J Gerontol* 46, P352-361. doi: 10.1093/geronj/46.6.p352

Bowen, D. J., Fesinmeyer, M. D., Yasui, Y., Tworoger, S., Ulrich, C. M., Irwin, M. L., et al. (2006). Randomized trial of exercise in sedentary middle aged women: effects on quality of life. *Int J Behav Nutr Phys Act* 3, 34. doi: 10.1186/1479-5868-3-34

Carcelén-Fraile, M. D. C., Aibar-Almazán, A., Martínez-Amat, A., Jiménez-García, J. D., Brandão-Loureiro, V., García-Garro, P. A., et al. (2022). Qigong for mental health and sleep quality in postmenopausal women: A randomized controlled trial. *Medicine (Baltimore)* 101, e30897. doi: 10.1097/MD.0000000000030897

Curi, V. S., Vilaça, J., Haas, A. N., and Fernandes, H. M. (2018). Effects of 16-weeks of Pilates on health perception and sleep quality among elderly women. *Arch Gerontol Geriatr* 74, 118–122. doi: 10.1016/j.archger.2017.10.012

Elavsky, S., and McAuley, E. (2007). Physical activity and mental health outcomes during menopause: a randomized controlled trial. *Ann Behav Med* 33, 132–142. doi: 10.1007/BF02879894

El-Sayed, A. A. M., and Ismail, M. M. (2022). Physico-chemodiversity variation between the most common calcareous red seaweed, Eastern Harbor, Alexandria, Egypt. *Heliyon* 8, e12457. doi: 10.1016/j.heliyon.2022.e12457

Farzane, A., and Koushkie Jahromi, M. (2022). The effect of pilates training on hormonal and psychophysical function in older women. *J Sports Med Phys Fitness* 62, 110–121. doi: 10.23736/S0022-4707.21.12089-4

Gao, L., Zhang, L., Qi, H., and Petridis, L. (2016). Middle-aged Female Depression in Perimenopausal Period and Square Dance Intervention. *Psychiatr Danub* 28, 372–378.

Gary, R., and Lee, S. Y. S. (2007). Physical function and quality of life in older women with diastolic heart failure: effects of a progressive walking program on sleep patterns. *Prog Cardiovasc Nurs* 22, 72–80. doi: 10.1111/j.0889-7204.2007.05375.x

Gusi, N., Reyes, M. C., Gonzalez-Guerrero, J. L., Herrera, E., and Garcia, J. M. (2008). Cost-utility of a walking programme for moderately depressed, obese, or overweight elderly women in primary care: a randomised controlled trial. *BMC Public Health* 8, 231. doi: 10.1186/1471-2458-8-231

Hu, L., Zhu, L., Lyu, J., Zhu, W., Xu, Y., and Yang, L. (2017). Benefits of Walking on Menopausal Symptoms and Mental Health Outcomes among Chinese Postmenopausal Women. *International Journal of Gerontology* 11, 166–170. doi: 10.1016/j.ijge.2016.08.002

Imayama, I., Alfano, C. M., Kong, A., Foster-Schubert, K. E., Bain, C. E., Xiao, L., et al. (2011). Dietary weight loss and exercise interventions effects on quality of life in overweight/obese postmenopausal women: a randomized controlled trial. *Int J Behav Nutr Phys Act* 8, 118. doi: 10.1186/1479-5868-8-118

Innes, K. E., and Selfe, T. K. (2012). The Effects of a Gentle Yoga Program on Sleep, Mood, and Blood Pressure in Older Women with Restless Legs Syndrome (RLS): A Preliminary Randomized Controlled Trial. *Evid Based Complement Alternat Med* 2012, 294058. doi: 10.1155/2012/294058

Kim, Y.-S., O’Sullivan, D. M., and Shin, S.-K. (2019). Can 24 weeks strength training reduce feelings of depression and increase neurotransmitter in elderly females? *Exp Gerontol* 115, 62–68. doi: 10.1016/j.exger.2018.11.009

Liu, Q. (2016). Effects of16 weeks Tai Chi exercise and stop practice 8 weeks impact on mental health of older women. *Front Public Health* 32, 99–103. doi: 10.3389/fpubh.2023.1295342

Luoto, R., Moilanen, J., Heinonen, R., Mikkola, T., Raitanen, J., Tomas, E., et al. (2012). Effect of aerobic training on hot flushes and quality of life--a randomized controlled trial. *Ann Med* 44, 616–626. doi: 10.3109/07853890.2011.583674

Ma, Z., Wang, B., and Xi, B. (2016). The effect of fitness Qigong·Mawangdui Daoyin exercises on the mood state and anxiety level of middle-aged and elderly women. *Chinese Journal of Gerontology* 36, 3248–9.

Newton, K. M., Reed, S. D., Guthrie, K. A., Sherman, K. J., Booth-LaForce, C., Caan, B., et al. (2014). Efficacy of yoga for vasomotor symptoms: a randomized controlled trial. *Menopause* 21, 339–346. doi: 10.1097/GME.0b013e31829e4baa

Noh, E., Kim, J., Kim, M., and Yi, E. (2020). Effectiveness of SaBang-DolGi Walking Exercise Program on Physical and Mental Health of Menopausal Women. *Int J Environ Res Public Health* 17, 6935. doi: 10.3390/ijerph17186935

Pang, Y., and Kim, O. (2021). Effects of Smartphone-Based Compensatory Cognitive Training and Physical Activity on Cognition, Depression, and Self-Esteem in Women with Subjective Cognitive Decline. *Brain Sci* 11, 1029. doi: 10.3390/brainsci11081029

Pinheiro, H. A., Cerceau, V. R., Pereira, L. C., Funghetto, S. S., and Menezes, R. L. de (2020). Nutritional intervention and functional exercises improve depression, loneliness and quality of life in elderly women with sarcopenia: a randomized clinical trial. *Fisioter. mov.* 33, e003332. doi: https://doi.org/10.1590/1980-5918.033.AO32

Sen, E. I., Esmaeilzadeh, S., and Eskiyurt, N. (2020). Effects of whole-body vibration and high impact exercises on the bone metabolism and functional mobility in postmenopausal women. *J Bone Miner Metab* 38, 392–404. doi: 10.1007/s00774-019-01072-2

Shahidi, M., Mojtahed, A., Modabbernia, A., Mojtahed, M., Shafiabady, A., Delavar, A., et al. (2011). Laughter yoga versus group exercise program in elderly depressed women: a randomized controlled trial. *Int J Geriatr Psychiatry* 26, 322–327. doi: 10.1002/gps.2545

Song, J., Wei, L., Cheng, K., Lin, Q., Xia, P., Wang, X., et al. (2022). The Effect of Modified Tai Chi Exercises on the Physical Function and Quality of Life in Elderly Women With Knee Osteoarthritis. *Front Aging Neurosci* 14, 860762. doi: 10.3389/fnagi.2022.860762

Soori, S., Heirani, A., and Rafie, F. (2022). Effects of the aerobic and Pilates exercises on mental health in inactive older women. *J Women Aging* 34, 429–437. doi: 10.1080/08952841.2021.1924576

Villaverde Gutiérrez, C., Torres Luque, G., Ábalos Medina, G. M., Argente del Castillo, M. J., Guisado, I. M., Guisado Barrilao, R., et al. (2012). Influence of exercise on mood in postmenopausal women. *J Clin Nurs* 21, 923–928. doi: 10.1111/j.1365-2702.2011.03972.x

Williams, P., and Lord, S. R. (1997). Effects of group exercise on cognitive functioning and mood in older women. *Aust N Z J Public Health* 21, 45–52. doi: 10.1111/j.1467-842x.1997.tb01653.x
